# A high-throughput dual system to screen polyphosphate kinase mutants for efficient ATP regeneration in L-theanine biocatalysis

**DOI:** 10.1186/s13068-023-02361-9

**Published:** 2023-08-03

**Authors:** Hui Gao, Mengxuan Li, Qing Wang, Tingting Liu, Xian Zhang, Taowei Yang, Meijuan Xu, Zhiming Rao

**Affiliations:** 1 The Key Laboratory of Industrial Biotechnology, Ministry of Education, School of Biotechnology, Jiangnan University, 1800 Lihu Road, Wuxi, 214122 Jiangsu China; 2 Yantai Shinho Enterprise Foods Co., Ltd., Yantai, 265503 China

**Keywords:** Polyphosphate kinase, ATP regeneration, High-throughput screening, Mutation

## Abstract

ATP, an important cofactor, is involved in many biocatalytic reactions that require energy. Polyphosphate kinases (PPK) can provide energy for ATP-consuming reactions due to their cheap and readily available substrate polyphosphate. We determined the catalytic properties of PPK from different sources and found that PPK from *Cytophaga hutchinsonii* (*Ch*PPK) had the best catalytic activity for the substrates ADP and polyP_6_. An extracellular–intracellular dual system was constructed to high-throughput screen for better catalytic activity of *Ch*PPK mutants. Finally, the specific activity of *Ch*PPK_D82N-K103E_ mutant was increased by 4.3 times. Therefore, we focused on the production of L-theanine catalyzed by GMAS as a model of ATP regeneration. Supplying 150 mM ATP, GMAS enzyme could produce 16.8 ± 1.3 g/L L-theanine from 100 mM glutamate. When 5 mM ATP and 5 U/mL *Ch*PPK_D82N-K103E_ were added, the yield of L-theanine was 16.6 ± 0.79 g/L with the conversion rate of 95.6 ± 4.5% at 4 h. Subsequently, this system was scaled up to 200 mM and 400 mM glutamate, resulting in the yields of L-theanine for 32.3 ± 1.6 g/L and 62.7 ± 1.1 g/L, with the conversion rate of 92.8 ± 4.6% and 90.1 ± 1.6%, respectively. In addition, we also constructed an efficient ATP regeneration system from glutamate to glutamine, and 13.8 ± 0.2 g/L glutamine was obtained with the conversion rate of 94.4 ± 1.4% in 4 h after adding 6 U/ mL GS enzyme and 5 U/ mL *Ch*PPK_D82N-K103E_, which further laid the foundation from glutamine to L-theanine catalyzed by GGT enzyme. This proved that giving the reaction an efficient ATP supply driven by the mutant enzyme enhanced the conversion rate of substrate to product and maximized the substrate value. This is a positively combination of high yield, high conversion rate and high economic value of enzyme catalysis. The mutant enzyme will further power the ATP-consuming biocatalytic reaction platform sustainably.

**Supplementary Information:**

The online version contains supplementary material available at 10.1186/s13068-023-02361-9.

## Introduction

Adenosine triphosphate (ATP) is an essential high-energy phosphate compound in living organisms [1]. It provides energy for processes such as synthesis, transportation, information transfer and so on in living cells [2]. ATP is usually used as a cofactor for group transfer in enzymatic catalysis, which is further involved in the production of high-value food chemicals (Additional file 1: Table S1). For example, some enzymes that produce glutamine [3], L-theanine [4] or glutathione [5] by biological methods require ATP as an energy molecule to participate in the reaction. S‑Adenosylmethionine (SAM), a donor of transmethyl, transaminopropyl and transsulfur, also directly requires ATP as a substrate. D-allulose is an effective substitute for sugar in food, which can be generated by transferring the phosphate group of ATP to D-fructose [6]. ATP energy drives the formation of amide bonds and catalyzes the reduction of carboxylic acids to aldehydes [7]. Aliphatic amines and carboxylic acid substrates produce amides and AMP under the action of ATP-dependent amide bond synthetase. In addition, the catalytic reactions from AMP to ATP may be more complex, with most involving two enzymes. Given the time and reaction cost of biological reactions, it does not seem feasible to add a large amount of additional ATP, which requires us to seek the sustainability and circularity of ATP supply methods.

At present, many enzymes have been reported to be used for ATP regeneration, such as pyruvate kinase (PK), creatine kinase (CK), acetate kinase (AK) and polyphosphate kinase (PPK) [8]. However, systems based on phosphoenolpyruvate (PEP), acetyl phosphate, and creatine may have problems with expensive, product inhibition or low stability, which limit the application of these technologies in large-scale production. It is preferred to use the cheap substrates, minimize the amount of enzyme in the process and the simple form of catalysis [9]. Inorganic polyphosphate (polyP) with a linear polymer of several phosphate has been found in all living cells containing archaea, bacteria, and eukaryote [10], serving as stress and survival, energy, cellular motility, biofilm formation [11] and a chelator of metal ions [12]. Recently, the reversible reaction of polyphosphate kinases (PPK) catalyzed by inorganic polyphosphate to ATP has attracted much attention. PPK can be divided into two families, PPK1 and PPK2 [13]. PPK1 tends to synthesize polyP using the terminal phosphate of ATP as substrate. Genetic and biochemical studies have identified the role of PPKs in polyP biosynthesis, such as *Escherichia coli*. PPK2s show the opposite characteristics which can catalyze polyP as a donor to adenosine phosphorylation [14]. PPK2s can be divided into three subfamilies (PPK2 I, PPK2 II, PPK2 III) [15]. PPK2 I prefer to convert ADP to ATP [16], PPK2 II may accept AMP as substrate for ATP regeneration [13] and PPK2 III have the ability to phosphorylate adenosine monophosphate and adenosine diphosphate at the same time [17].

In this work, we performed substrate profiling and substrate tolerance analysis for PPK from different sources and constructed a high-throughput dual system screening with intracellular and extracellular to screen the better enzyme activity of PPK-mutants. Molecular docking and structural analysis revealed the catalytic efficiency of the enzyme could be highly improved by enlarging the dual-substrate channel cavity. At the same time, this mutation could improve the utilization range of the substrate polyphosphates and the tolerance at different concentrations of polyphosphate. Therefore, based on this mutant enzyme, we developed a platform model for ATP regeneration to produce L-theanine and glutamine. With greatly reduced cost and broadened substrate selection range, this efficient ATP regeneration system will be further used in biocatalytic reactions.

## Materials and methods

### Strains, chemicals and culture condition

All strains and plasmids used or constructed in this work are listed in Additional file 1. The genes of *Bl*PPK (WP_112868569.1), *Cg*PPK (WP_074495911.1), *Pa*PPK2I (WP_003112634.1), *Pa*PPK2II (WP_070331057.1), *Pa*PPK2III (WP_003091935.1) were amplified from *E. coli*, *C. glutamicum*, *Pseudomonas aeruginosa*. The genes of *Eb*PPK from *Erysipelotrichaceae bacterium* [18] (HCY06753.1), *Ch*PPK from *Cytophaga hutchinsonii* (WP_011583516.1), *gmas* (MN514852.1) from *Methylovorus mays* were synthesized by GENEWIZ Bio Inc (Suzhou, China), respectively. The *glnA* (NZ_LOQW01000025.1) gene was amplified from the genome of *C. glutamicum*. All genes were introduced into the plasmid pET-28a ( +) or pXMJ-19. These recombinant plasmids were then transformed to *E. coli* BL21 (DE3). *E. coli* was grown in LB liquid medium-containing 5 g/L yeast extract, 10 g/L tryptone, and 10 g/L NaCl at 37 °C. LB solid medium was that 1.8 g agar were added per 100 mL of LB liquid medium. 50 μg/mL kanamycin or 10 μg/mL chloramphenicol were added to the LB medium. PolyP_3_, polyP_6_, and polyP_n_ (n > 10) were purchased from Aladdin (Shanghai, China) Co., LTD. Hexokinase and glucose-6-phosphokinase were purchased from Merck Sigma-Aldrich Co., LTD. L-Glutamate (BC Grade, > 99%), L-cysteine (Reagent Grade, 98.5%), L-glycine (BC Grade, 98.5%), and glutathione (Reagent Grade, 98.5%) were purchased from Sangon Biotechnology (Shanghai, China) Co., LTD. The random mutation kit was purchased from Beijing TIANDZ Biotechnology Co., LTD., and the other reagents were purchased from Sinopharm Chemical Regent Co., LTD. Prices for all chemicals in the manuscript refer to Macklin (Shanghai, China).

### Protein overexpression and purification

The recombinant cells were incubated in 10 mL LB medium at 37 ℃ for 12 h. Then, 1% cultures were transferred to 50 mL of LB medium in 250-mL flasks and cultivated at 37 ℃. PPK, glutamylmethylamide synthetase (GMAS) and glutamine synthetase (GS) overexpression was induced by adding 0.2 mM isopropyl-β-d-thiogalactoside (IPTG) until the *OD*_600_ achieved 0.7, and then the cells were incubated at 16 ℃ and 220 r/min for 18 h [19]. The cells were suspended in pH 7.4, 200 mM PBS buffer and lysed by sonication (15 min at 4 ℃) on ice to release intracellular proteins. The resulting suspension was centrifuged at 11,100 g for 25 min at 4℃ and the liquid supernatant were collected. The supernatant was applied to a Ni^2+^-NTA column to get the purified enzymes. Protein purification was performed as described previously [20]. The purified proteins were analyzed via SDS-PAGE. Protein concentration was determined by the Bradford method [21].

### Enzyme activity and kinetic parameters

Hexokinase (HK) is responsible for catalyzing the first step in glycolysis. It causes glucose and ATP to form glucose-6-phosphate and ADP. Glucose-6-phosphate dehydrogenase (G6PD) is a key enzyme in the pentose phosphate pathway and can convert glucose-6-phosphate and NADP to NADPH and gluconolactone 6-phosphate[22]. The enzyme activity of PPK was determined by HK and G6PD [23]. The reaction mixtures (2 mL) contained the final concentrations of 100 mM Tris–HCl (pH 8.0), 10 mM glucose, 10 mM NADP, 25 mM MgCl_2_, 10 mM ADP, 50 mM polyP_6_, 5 U HK, 5 U G6PD and enzyme. The reaction was initiated by the addition of polyP_6_. To verify the effects of polyP-chain length, we select polyP_3_, polyP_6_, polyP_n_ (*n* > 6) in reaction mixtures. 50 mM, 100 mM, 150 mM and 200 mM polyP_6_ were applied for exploring the effects of polyP_6_ concentration. The mixture containing 10 mM ADP or 10 mM AMP were also analyzed. The reaction was incubated at 35℃ for 30 min and the mixture was measured at 340 nm for absorbance value. One unit of PPK (1 U) is defined as the amount of enzyme required to produce 1 μmol ATP per minute.

The *K*_m_ and *V*_max_ values of the enzyme were determined by double reciprocal method [24]. The *K*_m_ and *V*_max_ values of the WT, D82N and K103E were measured of 100 mM Tris–HCl (pH 8.0) with different concentrations of ADP (0.1 to 5 mM) and polyP_6_ (1 to 7.5 mM), respectively. The kinetic parameters of ADP and polyP_6_ were determined by the enzyme activity method described above. All experiments were performed in triplicate.

Enzyme activity of GS was determined as described previously [3]. The reaction solution was centrifuged at 15,984 g for 5 min, and the absorbance value of the supernatant was measured at 540 nm. One unit of GS (1 U) is defined as the amount of enzyme required to produce 1 μmol γ-glutamyl hydroxamic acid per minute.

### Creation of the mutant library by error-prone PCR

The error-prone PCR of *chppk* gene was performed by TIANDZ error-prone PCR kit (https://tiandz.etlong.com/). The plasmid pXMJ-19-tac-*Ch*PPK was used as template, and the F and R primers are seen Additional file 1 and were applied to obtain the mutant fragments of *Ch*PPK from error-prone PCR, accompanied by the changes of MgCl_2_ and MnCl_2_ [25]. The fragments were ligated to the plasmid pXMJ-19-*rrnB* P1-GFP and then transformed to the *E. coli* BL21 (DE3). The transformed strains were spread on LB-agar plates containing 10 μg/mL chloramphenicol and incubated at 37 ℃ for 24 h for obtaining transformants (BL21/pXMJ-19-tac-*Ch*PPKmuts- *rrnB* P1-GFP).

### Construction of extracellular–intracellular dual system to high-throughput screen

The extracellular high-throughput screening method is based on the chromogenic reaction. It was reported that 3,3′,5,5′-tetramethylbenzidine (TMB) has a benzidine structure, which contains two amidogens that are easy to oxidize. ATP has the property of peroxidase, which can catalyze the oxidation of TMB by H_2_O_2_ under weak acidic conditions [26]. The oxidized TMB solution appears blue and has an absorbance value at 652 nm [27]. The transformants mentioned above (BL21/pXMJ-19-tac-*Ch*PPKmuts-*rrnB* P1-GFP) were cultured in 24-well plates containing 3 mL LB medium for 12 h at 37 ℃, and then 1% of the inoculum were transferred to the new 24-well plate that cultured at 37℃ for 2 h. 0.5 mM IPTG were added to the 24-well plate for induction at 16 ℃ for 12 h. The culture mediums were washed twice with PBS buffer (pH 7.4), and then the cells were collected for subsequent experiments. The mutants with high enzyme activity were screened by TMB-H_2_O_2_-ATP chromogenic method [28]. First, 1 mL Tris–HCl (pH 8, 50 mM) of the solution A contained 10 mM polyP_6_, 5 mM MgCl_2_, 10 mM ADP and the collected cells. The solutions were reacted at 35 ℃ for 1 h. After centrifugation at 15,984 g for 1 min, 400 uL of the supernatant was taken and added to the solution B of 200 mM PBS buffer (pH 5.0) containing 5 mM TMB, 100 mM H_2_O_2_ at 40 ℃ for 30 min. The reaction solutions were terminated with 10% trichloroacetic acid. The solutions were screened for mutants with improved enzyme activity by detecting the absorbance value at 652 nm. In order to make the reaction more sensitive to the detection of ATP, we first optimized the concentration of TMB and H_2_O_2_. We observed the effects of different concentrations of TMB (1 mM, 5 mM, 10 mM) and H_2_O_2_ (50 mM, 100 mM, 200 mM) on the absorbance values of different concentrations of ATP (0.1 mM, 0.5 mM, 1 mM, 2.5 mM, 5 mM).

The activity of the ribosomal RNA promoter *rrnB* P1 has been shown to depend on ATP concentration in *E. coli* cells [29]. RNA polymerase holoenzymes and *rrnB* P1 form a very short active complex that requires high concentrations of ATP to initiate transcription of rRNA. We used the strains BL21/pXMJ-19-tac-*Ch*PPKmuts-*rrnB* P1-GFP to detect the fluorescence value. The mutants were selected and cultured in 24-well plates at 37 ℃ for 12 h. 1% of the inoculum were transferred to the new 24-well plate that cultured at 37 ℃ for 2 h. 0.5 mM IPTG, 5 mM ADP and 8 mM polyP_6_ were added to the 24-well plate for induction at 16 ℃ for 12 h. The strains were collected and were suspended in PBS buffer. The fluorescence value was detected by Elisa, with the *OD*_600_ value. We selected mutants with high fluorescence value/*OD*_600_ value for subsequent experiments, compared with the wild type.

### Site-directed saturation mutation

Residues D82 and K103 in wild-type *Ch*PPK were mutated to the remaining 20 amino acids by site-directed PCR [30], respectively. Site-directed mutagenesis was accomplished by designing primers containing the specific mutant sequences, which are shown in Table S3. The pXMJ-19-*chppk* was used as the DNA template. The plasmids were transformed into *E. coli* BL21 (DE3) and spread on LB-agar plates containing 10 μg/mL chloramphenicol and incubated at 37 ℃ for 24 h. The mutants with correct DNA sequencing were selected for enzyme activity analysis.

### Structural analysis of *Ch*PPK and mutants

The PDB ID of the protein *Ch*PPK was 6AN9. Docking between substrate and protein was completed by Schrödinger-glide dock and the best docking position was selected by docking analysis. Import the docking box and ligand, set the docking accuracy to SP (standard precision). The root mean square deviation (RMSD) reflects the position change degree of the molecular structure with time (shift degree), while the root mean square deviation (RMSF) represents the freedom degree of movement of each atom in the molecule [31]. RMSD and RMSF were performed by using GROMACS [32]. MD simulations were performed using GROMACS 2019.623 and GROMOS96 force fields 24. Each system is immersed in a dodecahedral tank of spc216 dominant water, and steps such as energy minimization, system balancing, and production protocols are performed after neutralizing the charge of the system. 2 ns canonical ensemble (NVT) and constant-pressure, constant-temperature (NPT) were run at 300K with a step size of 2 fs. Finally, a 30-ns simulation was run at 300K with a step size of 2 fs. The software Pymol (Pymol Molecular Graphics System, 2.0 Schrödinger, LLC) was used to visualize the proteins.

### Determination for the ability to regenerate ATP

Systems for the ATP-producing capacity of PPK enzymes (200 mL) included Tris–HCl (100 mM, pH 8), 20 g/L polyP_6_, 20 g/L ADP, 10 g/L MgCl_2_ and the pure enzymes (5 U/mL). The reaction was at 37 ℃, 150 r/min in the shaker for 24 h. Samples were placed in boiling water for 5 min to terminate the reactions and measured the production of ATP.

### Construction of ATP-regeneration platform

System of L-theanine production (200 mL) by GMAS enzyme consists of Tris–HCl (100 mM, pH 8.0), 100/200/400 mM L-glutamate, 100/200/400 mM ethylamine, 200 mM (NH_4_)_2_SO_4_, 50 mM polyP_6_, 5 mM ATP, 50 mM MgCl_2_, 5 U/mL PPK, 10 U/mL GMAS. The reaction was at 37 ℃, 150 r/min in the shaker for 24 h. System of glutamine production (200 mL) by GS enzyme consists of Tris–HCl (100 mM, pH 8.0), 100 mM L-glutamate, 100 mM (NH_4_)_2_SO_4_, 50 mM polyP_6_, 5 mM ATP, 50 mM MgCl_2_, 5 U/mL PPK, 6 U/mL GS. The reaction was at 37 ℃, 150 r/min in the shaker for 24 h. The samples were placed in boiling water for 5 min to terminate the reactions. Samples were centrifuged at 15,984 g for 5 min and measured the yield by HPLC.

### Analytical methods

Glutamine, L-theanine, glutamate and ethylamine were measured by HPLC with the method of precolumn derivatization o-phthaldialdehyde (OPA) [3, 4]. The chromatographic conditions were as follows: C18 column (250 × 4.6 mm, 5 μm), 40 °C, 1.0 mL/min, and the wavelength was 338 nm.

## Results and discussion

### Screening and characterization of PPK enzymes

**Fig. 1:**
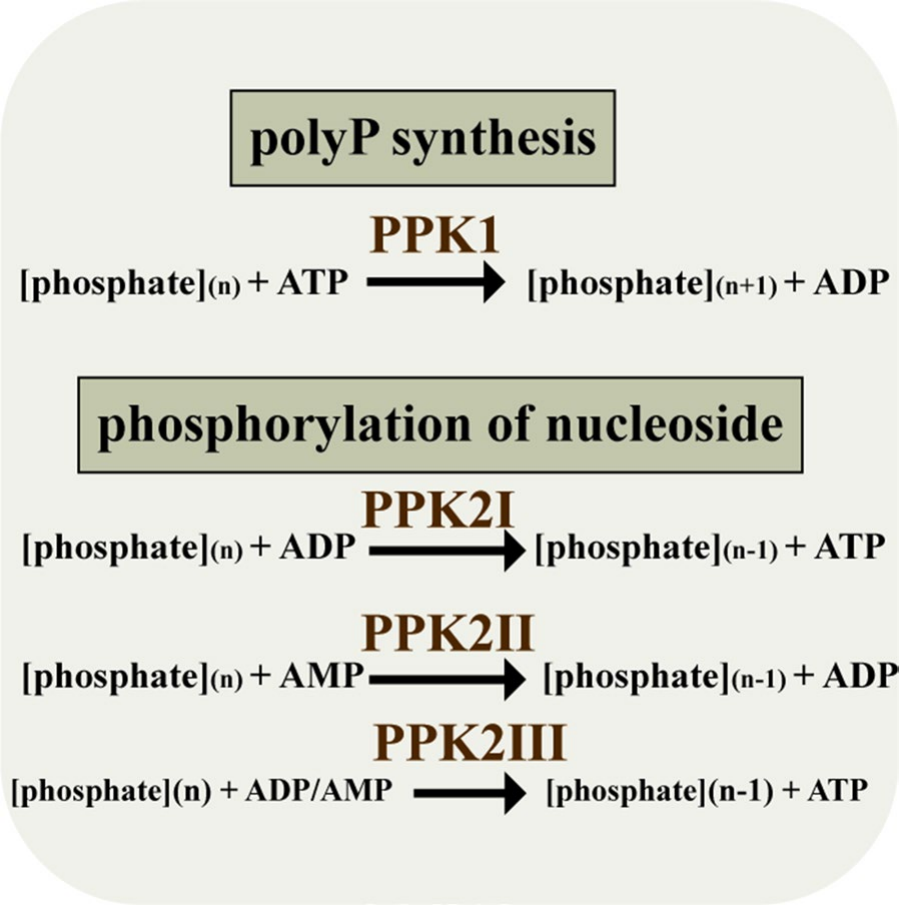
Catalytic direction of PPK enzymes

PPK enzymes can be divided into two major groups: PPK1 and PPK2. PPK1 can convert ATP into polyP, and PPK2 can convert ADP or AMP to ATP using polyP as a donor (Fig. 1) [16]. In order to explore the properties of PPK enzymes from different sources, we designed an evolutionary tree of PPK enzymes from NCBI (Additional file 1: Fig. S1). PPKs from *Escherichia coli* (*Bl*PPK), *Corynebacterium glutamicum* (*Cg*PPK), *Pseudomonas aeruginosa* (*Pa*PPK2I, *Pa*PPK2II, *Pa*PPK2III), *Erysipelotrichaceae bacterium* (*Eb*PPK), *Cytophaga hutchinsonii* (*Ch*PPK) were selected and all genes were introduced into the plasmid pET-28a. The plasmids have been successfully expressed in *E. coli* BL21(DE3). SDS-PAGE analysis indicated that the five PPKs were expressed successfully (Additional file 1: Fig. S2). The expression of *Eb*PPK and *Ch*PPK on the pET-28a plasmid may be due to the fast translation of the proteins, the proteins were clustered together (Additional file 1: Fig. S2). *Eb*PPK and *Ch*PPK were failed to purify (data not shown). Therefore, *EB*PPK and *Ch*PPK were chose to express on the pXMJ-19 plasmids. The results showed that the two proteins were expressed clearly and purified successfully (Additional file 1: Fig. S2).

**Fig. 2:**
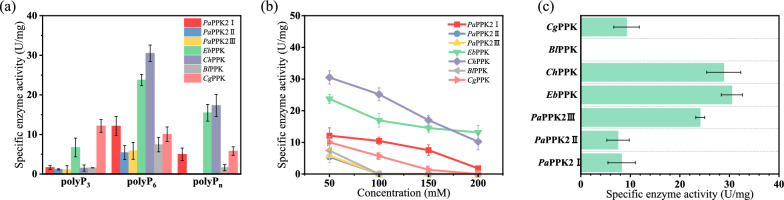
Catalytic properties of the PPK enzymes. **a** Effect of phosphate-chain length on enzyme activity. **b** Effect of polyP_6_ concentration on enzyme activity. **c** Catalytic activity of PPK enzyme on the substrate AMP

As the catalytic performance of the enzyme depends on the substrates and concentration [33, 34], the length and concentration of polyP were performed to analyze the catalytic function of PPKs in this work. After the seven PPK enzymes were purified by Ni^2+^-NTA column, the purified enzymes were added to the system in "Enzyme activity and kinetic parameters" section. First, 50 mM polyP_3_, polyP_6_ and polyP_n_ were carried out to show the specific activities of the seven PPK enzymes. In general, the polyP_6_, rather than polyP_3_ and polyP_n_, was responsible for the better catalytic capacity of PPK enzymes and the *Ch*PPK was termed as the best biocatalyst for polyP_6_ to ATP regeneration with 30.26 U/mg (Fig. 2a).

Almost all PPK hardly indicated enzyme activity with polyP_3_ except *Cg*PPK and *Eb*PPK. The short phosphate-chain (polyP_3_) may block the entrance of ADP binding pocket for the lower enzyme activities. Due to the sensitivity of PPK to phosphate-chain length [34], the effects of polyP_6_ at different concentrations on the enzyme activity were proposed. The ability of ATP formation was gradually inhibited with the increase of polyP_6_ concentration (Fig. 2b). *Pa*PPK2II, *Pa*PPK2III and *Bl*PPK explored no catalytic activity at polyP_6_ concentration of 100 mM, 150 mM and 200 mM. *Ch*PPK showed the best enzyme activity at 50 mM, 100 mM and 150 mM polyP_6_. When the concentration of substrate polyP_6_ was 200 mM, the specific activity of *Ch*PPK was dropped by 66.5%, and the specific activity was 10.23 U/mg. In view of the exploration to the catalytic properties of the seven enzymes, *Ch*PPK was selected for subsequent experiments, and molecular modification targeting *Ch*PPK is necessary to improve the tolerance of *Ch*PPK to polyP_6_ and enhance the ATP supply capacity.

In addition, some PPK enzymes can catalyze from AMP to ATP, so we decided to explore the effect of AMP on the activity of PPK enzymes (Fig. 2c). *Eb*PPK was capable of the best catalytic efficiency for converting AMP, followed by *Ch*PPK and *Pa*PPK2III. The unique catalytic activity on AMP is due to the fact that these enzymes belong to PPK2 class III [18]. This also provides the basis for the subsequent transfer and regeneration of two phosphate groups between ATP and AMP.

### Construction of the high-throughput screening method

In order to further improve the enzyme activity of *Ch*PPK and its tolerance to the substrate phosphate polyP, we screened *Ch*PPK mutants that could efficiently use polyP in the ATP regeneration system to enhance the supply of ATP in the biocatalytic process. In this research, a high-throughput screening method was used to screen *Ch*PPK mutants with excellent catalytic performance. We constructed the plasmid pXMJ-19-tac-*Ch*PPK and error-prone PCR was chosen to access mutation fragments of the gene for *Ch*PPK. Finally, we constructed a mutant library containing 1008 strains (BL21/pXMJ-19-tac-*Ch*PPKmuts-*rrnB* P1-GFP).

**Fig. 3:**
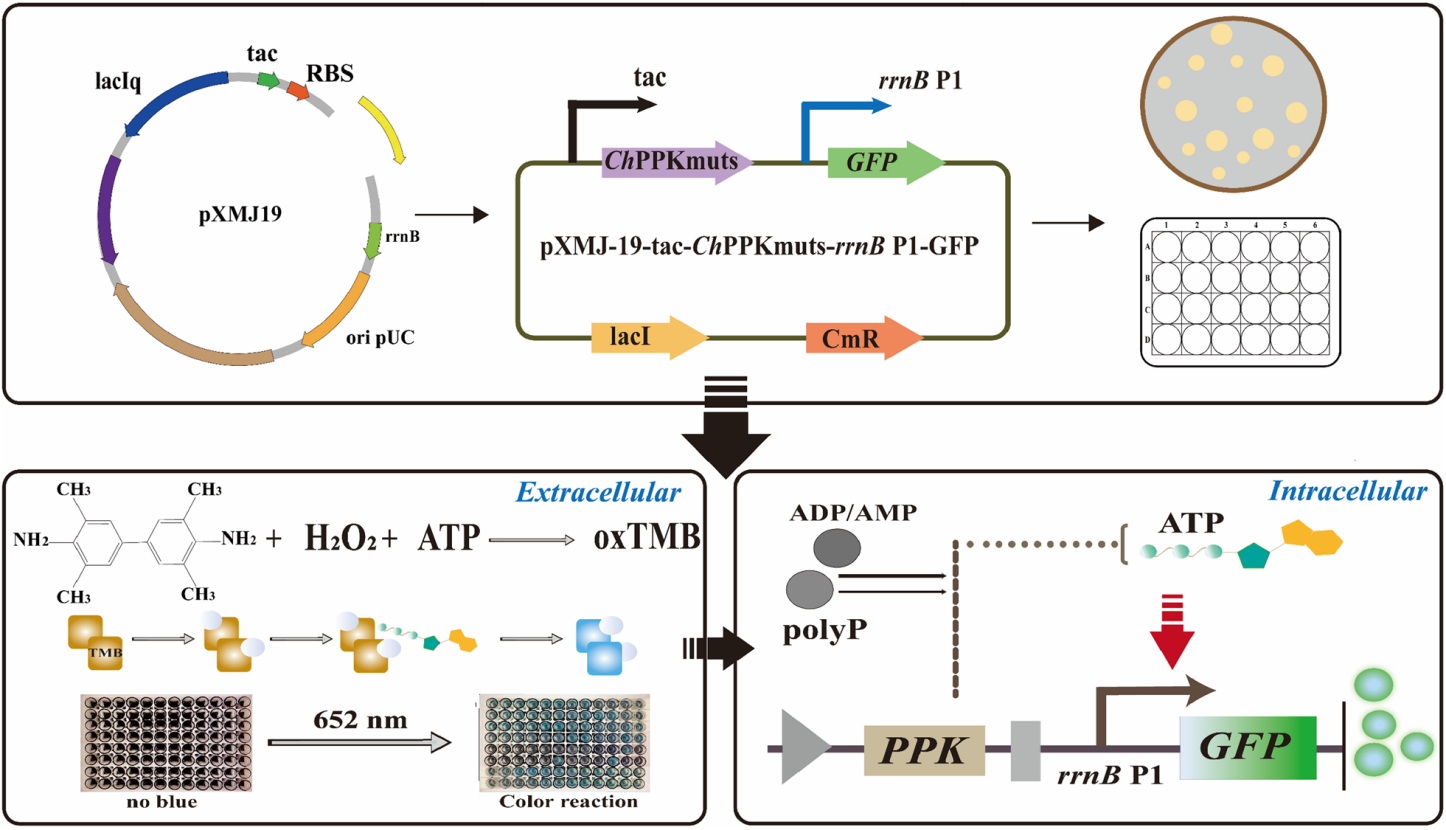
Flowchart of high-throughput screening

First, the extracellular high-throughput screening method is based on the TMB-H_2_O_2_-ATP chromogenic method (Fig. 3). TMB has a benzidine structure, which contains two amino compounds that are prone to oxidation. ATP has the property of peroxidase and can catalyze the oxidation of TMB by H_2_O_2_ under weakly acidic conditions; the oxidized TMB solution is blue and has an absorbance value at 652 nm. Based on this property, we constructed an ATP-TMB-H_2_O_2_ extracellular high-throughput screening method to screen the *Ch*PPK mutants with improved enzyme activity.

Considering that ATP is produced in the cell, there may still be some ATP in the cell due to intracellular and extracellular mass transfer, we also constructed an intracellular high-throughput screening method to verify the bidirectional screening of extracellular and intracellular ATP. It has been shown that expression of *rrnB* P1 (the ribosomal RNA promoter) is related to intracellular ATP content in* E. coli* and that RNA polymerase holoenzyme and *rrnB* P1 form a very short active complex that requires ATP to initiate transcription of rRNA. We constructed the plasmid pXMJ-19-tac-*Ch*PPKmuts-*rrnB* P1-GFP to verify whether the fluorescence intensity of GFP protein under the *rrnB* P1 promoter was responsive to the concentration of ATP (Fig. 3). The intracellular ATP concentration was reflected by measuring the GFP fluorescence intensity of the mutants.

### High-throughput screening of *Ch*PPK mutants by extracellular–intracellular dual system

**Fig. 4:**
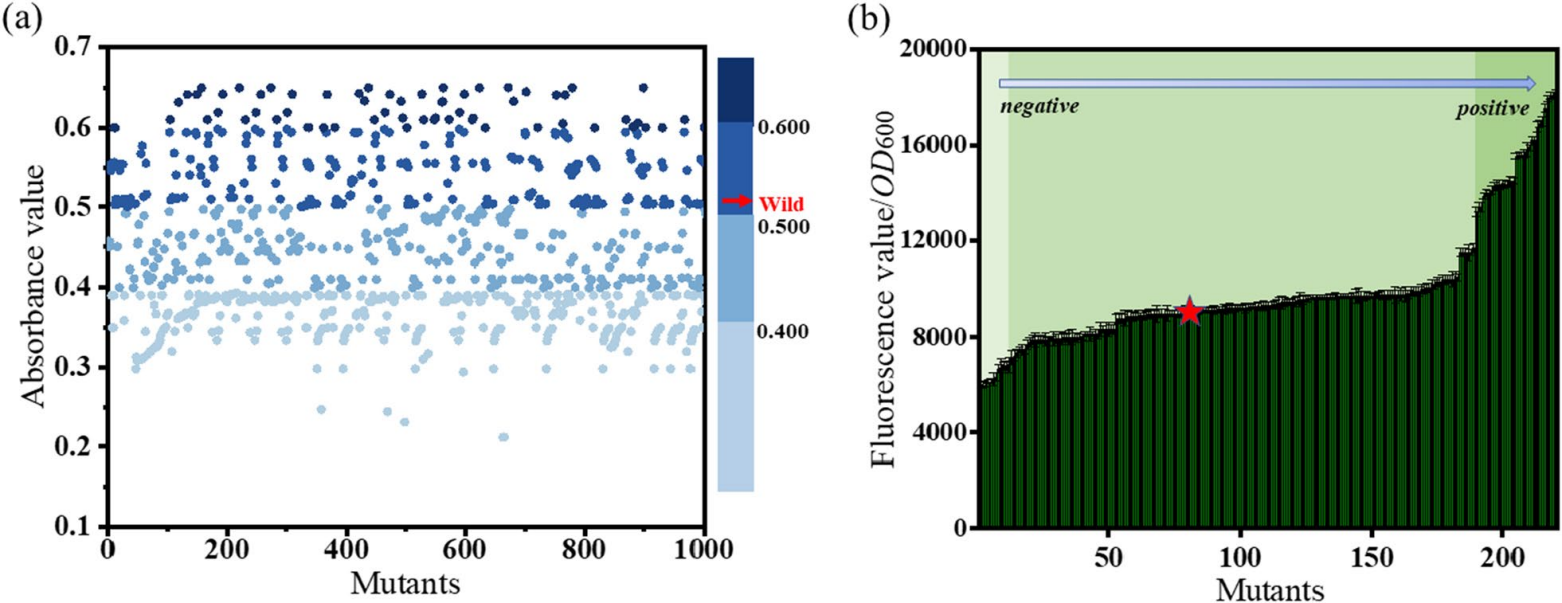
Results of the high-throughput screening of *Ch*PPK mutants. **a** Results of the extracellular screening, dark blue: positive mutants, light blue: negative mutants. **b** Results of the fluorescence screening, the red star mark: the wild strain

First, the extracellular high-throughput screening method is based on the TMB-H_2_O_2_-ATP chromogenic method. To make ATP concentration more sensitive to the response of the color reaction, the extracellular screening system of TMB and H_2_O_2_ were optimized. After optimization, 5 mM TMB and 100 mM H_2_O_2_ were selected for subsequent experiments (Additional file 1: Fig. S3). By detecting the absorbance values of the 1008 strains at 652 nm, the absorbance value of wild-type *Ch*PPK at 652 nm was 0.512, and 188 mutants were finally screened, which had absorbance values higher than 0.512, and these mutants may have better catalytic activity compared to the wild-type (Fig. 4a).

An ATP-GFP sensor in cells was used to further analyze the higher enzyme activity among 188 positive-mutants. Firstly, BL21/pXMJ-19-*rrnB* P1-GFP (EB10) was constructed to verify the effect of different ATP concentrations (0–10 mM) on fluorescence values. With the increase of ATP concentration, the fluorescence value/*OD*_600_ value gradually increased (Additional file 1: Fig. S3). We selected 188 positive-mutant strains from the extracellular screening library, and randomly screened 12 negative-mutant strains in the extracellular high-throughput results to detect their fluorescence intensity and the wild type was used as a control. Compared to the wild strain, the 30 mutant strains with a range of 10.4% to 55.9% improvement showed higher fluorescence values and 12 negative-mutant strains presented predictably lower fluorescence values (Fig. 4b).

**Fig. 5:**
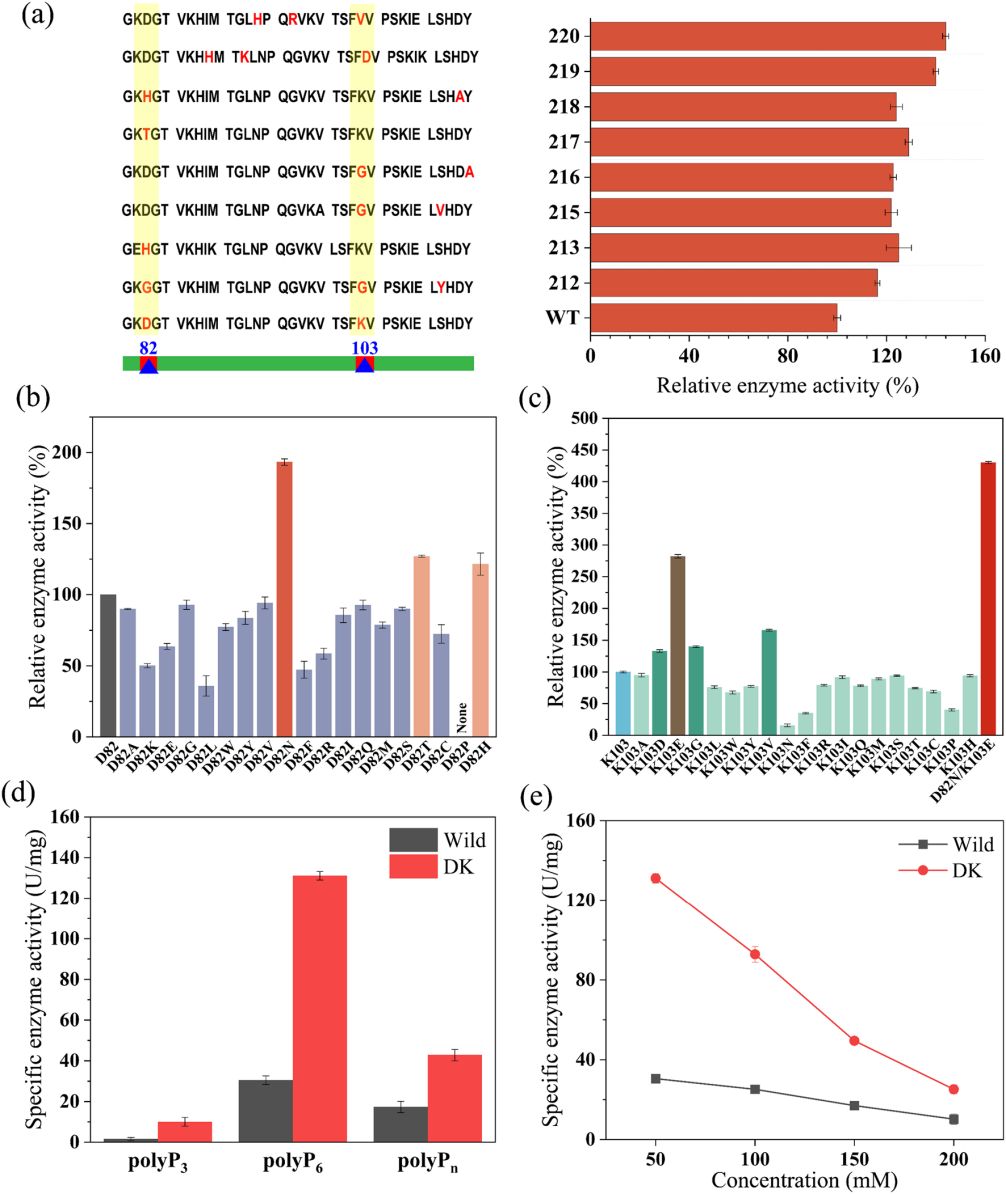
Enzyme activity assay of mutants and characterization of the *Ch*PPK_D82N-K103E_. **a** Relative enzyme activity of the positive mutants. **b** Relative enzymatic activity of the 82-residue saturated mutants. **c** Relative enzymatic activity of the 103-residue saturated mutants and the DK mutant. **d** Effect of phosphate-chain length on enzyme activity of the mutant strain DK. **e** Effect of polyP_6_ concentration on enzyme activity of the mutant strain DK

The 30 mutants were induced to express the protein *Ch*PPK. The results suggested that the enzyme activity of most mutants was similar or slightly higher than that of wild type (data not shown). In particular, the enzyme activity of the eight mutants increased significantly (Fig. 5a). DNA sequencing of the mutant plasmids revealed that most plasmids contained mutations in residues D82 and K103 (changes: D82H, D82T, D82G, K103V, K103D, K103G) (Fig. 5a). Therefore, we suspected that D82 and K103 may be the key residue sites that affect the activity of *Ch*PPK enzyme. The sites of D82 and K103 were applied for the further experiments to construct the saturated mutation libraries.

### Directional saturation mutation and catalytic properties of mutants

After measuring the enzyme activity of the saturated mutant library, the mutant D1 (D82N) obtained the highest enzyme activity with the 93.3% improvement in the saturated mutation library of D82, while D2 (D82H) and D3 (D82T) also improved the 21.5% and 27.0% of enzyme activity compared the wild mutant (Fig. 5b). And the mutant K1 (K103E) showed the 282.2% of enzyme activity compared to the wild mutant with K2 (K103V), K3 (K103G) and K4 (K103D) displaying 166.0%, 140.0% and 133.0%, respectively (Fig. 5c). Then we constructed the double-mutant DK (D82N/K103E) and the enzyme activity of this mutant increased to 430.2% with the best catalytic ability (Fig. 5c).

As previously mentioned, the wild type of *Ch*PPK almost had no enzyme activity for polyP_3_. Thus, we analyzed the catalytic properties of the mutant DK. In particular, the relative enzyme activities of the substrates polyP_3_, polyP_6_ and polyP_n_ for *Ch*PPK_D82N-K103E_ improved by 6.6, 4.3 and 2.5 times, respectively (Fig. 5d). At the same time, the mutant also showed increased tolerance to polyP_6_. At the concentration of 50 mM polyP_6_, the enzyme activity of *Ch*PPK_D82N-K103E_ increased by about 4.3 times, while at the concentration of 100 mM, 150 mM and 200 mM, the relative enzyme activity increased by 3.7, 2.9 and 2.5 times (Fig. 5e). Notably, the double-mutant DK showed the best positive effect on the enzyme activity for the different substrates and tolerance.

In addition, we also determined the kinetic parameters of the pure enzyme for substrates ADP and polyP_6_ in wild-type and mutant strains (Table 1). The *K*_cat_/*K*_m_ values of K103E and D82N to the substrate ADP were 3.4 and 2.2 times higher than those of wild type. This indicated that K103E and D82N increased the affinity of ADP. However, for the substrate polyP_6_, the *K*_cat_/*K*_m_ value of K103E did not increase much while of D82N increased by 1.5-fold, confirming that the interaction between N82 and polyP_6_ was enhanced.

**Table 1:** The *K*_m_ and *K*_cat_ values of the mutants to substrate ADP and polyP_6_

Substrates	Strains	*K*_m_ (mM)	*K*_cat_ (S^−1^)	*K*_cat_/*K*_m_ (S^−1^ M^−1^)
ADP	WT	1.39 $\pm 0.$07	10.04 $\pm$ 0.13	7.22 $\times {10}^{3}$
ADP	K103E	0.81 $\pm 0.$12	19.69 $\pm 0.12$	24.3 $\times {10}^{3}$
ADP	D82N	0.95 $\pm 0.$03	15.05 $\pm 0.04$	15.8 $\times {10}^{3}$
polyP_6_	WT	2.3 $\pm$ 0.02	35.16 $\pm 0.19$	15.3 $\times {10}^{3}$
polyP_6_	K103E	2.24 $\pm$ 0.02	37.17 $\pm$ 0.02	$17.0\times {10}^{3}$
polyP_6_	D82N	2.12 $\pm$ 0.01	49.24 $\pm$ 0.13	23.2 $\times {10}^{3}$

### The mechanism of active sites for mutants and analysis by molecular docking and MD simulation

**Fig. 6:**
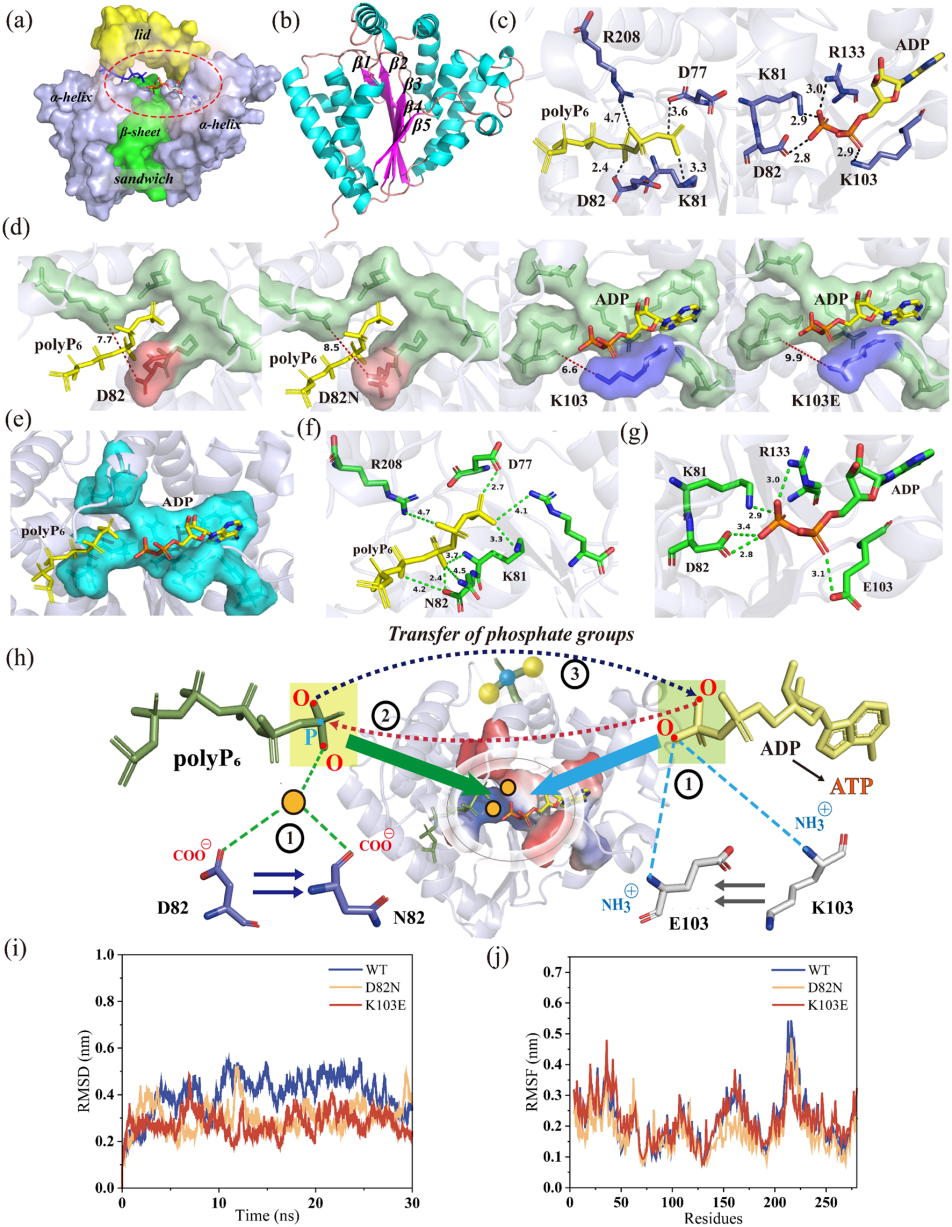
Analysis of the active-channel for *Ch*PPK. **a** The structure of *Ch*PPK enzyme. **b** The β-sheet of the *Ch*PPK protein structure. **c** Interaction of substrate polyP_6_ and ADP with residues. **d** Changes of substrate channels before and after mutation. **e** Changes in the dual-substrate channel after mutation. **f** Effects of the residue 82 mutation on active channel. **g** Effects of the residue 103 mutation on active channel. **i** Comparison of RMSD between the wild type and mutants. **j** Comparison of RMSF between the wild type and mutants

*Ch*PPK interacts with the substrates polyP_6_ and ADP in the active sites. Residues around the active-channel have an important effect on enzyme activity [35]. The protein structure of *Ch*PPK (PDB: 6AN9) and the molecules of polyP_6_ and ADP were docked by Schrodinger. The *Ch*PPK protein is composed of a structure like a "sandwich" (Fig. 6a). The left and right sides of the protein are both α-helix chains, and the middle is wrapped with a β-sheet (β1, β2, β3, β4, β5) (Fig. 6b). Above the "sandwich", there is a helix structure like a lid. The middle of the "sandwich" body and the lid form a cavity which is the site of phosphate group transfer between the substrate polyP_6_ and ADP (Fig. 6a). PolyP_6_ associates with D77, K81, D82 and R208 (3.6 Å, 3.3 Å, 2.4 Å and 4.7 Å), while ADP forms interaction of ions with residues K81, D82, K103 and R133 (2.9 Å, 2.8 Å, 2.9 Å and 3.0 Å) (Fig. 6c). D82 and K103 residues are located at the entrance of polyP_6_-channel and ADP-channel, respectively, and form ion interactions with the phosphate groups of the substrates. The distance between the two residues at the entrance of the polyP_6_-channel was calculated. The distance between R208 and D82 residue was 7.7 Å, and between R208 and D82N residue was 8.5 Å (Fig. 6d). Then, at the entrance of ADP-channel, the distance between K103E and D82 changes from 6.6 Å to 9.9 Å (Fig. 6d). The enhancement of enzyme activity by mutation is associated with the expansion of the active channel (Fig. 6e). D82N and K103E widen the entrances of polyP_6_ and ADP channels, respectively, and more substrates can enter the active center. This is the first time that the enzyme activity of polyphosphate kinase has been improved by mutation to enlarge the substrate-channel cavity.

Residues near the active center of PPK2s form connection with Mg^2+^ and substrates and then nucleophilic attack between substrates promotes the transfer of phosphate groups to form ATP [36]. There are two main ways of ionic interaction between residues and substrates, amino groups interacting with oxygen atoms or carboxyl groups interacting between magnesium ions and oxygen atoms (Fig. 6h). With the effect of Mg^2+^, the carboxyl group of the residue N82 is connected to the oxygen of the phosphate group from the polyP_6_ (2.4 Å, 4.2 Å, 4.5 Å) (Fig. 6f). The amino group of the residue K103E is directly related to the oxygen atom of ADP at the distance of 3.1 Å (Fig. 6g).

Then, we analyzed the flexibility of this mutant protein by RMSD and RMSF. The results of RMSD showed that the system fluctuation of WT, D82N and K103E mutants was stable and the molecular trajectories are in equilibrium (Fig. 6i). The RMSF of D82N and K103E were increased with the improvement of the flexibility of molecular structures (Fig. 6j). The fluctuation of the residues D82N and K103E indicated the flexibility of the pocket increased, which improved the interaction between the substrates and the pocket binding proteins.

### Determination for the ability of the mutant enzyme to regenerate ATP

More biocatalytic reactions require ATP to provide energy and it is increasingly necessary to construct a biological reaction platform for ATP regeneration. Based on the ATP regeneration platform of the *Ch*PPK_D82N-K103E_ enzyme, we verified the ability of this mutant enzyme to produce ATP compared to the other PPK enzymes described above. The results demonstrated that the purified mutant enzyme could convert 46.8 mM (20 g/L) ADP into 44±0.4 mM (18.8±0.19 g/L) ATP and the time-space yield of ATP was 4.7±0.05 g/L/h and exhibited the best ability of ATP production compared to the other six enzymes (Additional file 1: Table S3).

### Construction of the sustainable ATP-regeneration platform

**Fig. 7:**
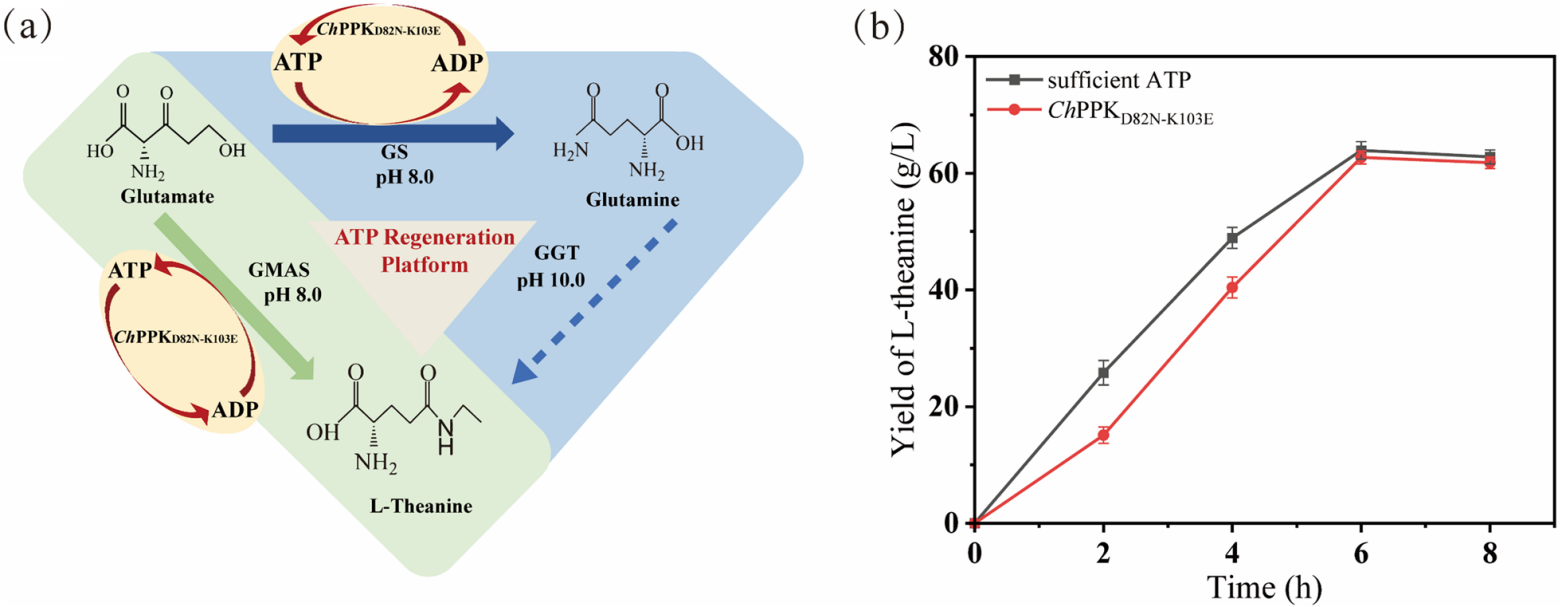
Application of the ATP regeneration platform to produce L-theanine. **a** Enzymatic synthesis of L-theanine. **b** Yield of L-theanine produced by enzyme catalysis (400 mM glutamate, 400 mM ethylamine and 5 mM ATP provided)

To verify the improved effect of *Ch*PPK_D82N-K103E_ for ATP regeneration and reduce the cost in enzymatic catalysis, we focused on the GMAS enzyme to produce L-theanine as the model of ATP regeneration platforms (Fig. 7a). GMAS can accept glutamate, ethylamine and ATP as the substrates. We constructed the strain EB14 (BL21/pET-28a-GMAS). GMAS and *Ch*PPK_D82N-K103E_ were purified by inducing the expression of strain EB14 and strain DK, respectively. GMAS and *Ch*PPK_D82N-K103E_ were added to the reaction system described in "Construction of ATP-regeneration platform" section. As shown in Additional file 1: Table S4, provided sufficient ATP (150 mM), the GMAS could convert 100 mM glutamate to 96.4±7.5 mM (16.8±1.3 g/L) L-theanine in 4 h. While only 5 mM ATP and 5 U/mL *Ch*PPK_D82N-K103E_ were added, L-theanine was produced 95.6±4.5 mM (16.6±0.79 g/L) at 4 h under the combined action of GMAS and *Ch*PPK_D82N-K103E_. Compared to the wild enzyme *Ch*PPK, the yield of the L-theanine improved 53.7%. *Ch*PPK_D82N-K103E_ was responsible for the similar yield of L-theanine to that produced by adding sufficient ATP in the substrates. Subsequently, the reactions were scaled to 200 mM glutamate in 200-mL reaction system, respectively. *Ch*PPK_D82N-K103E_ still revealed the similar ATP supply capacity. The yield of L-theanine was reached to 32.3±1.6 g/L with 92.8±4.6 % substrates converted within 6 h. Finally, we expanded the substrate concentration to 400 mM to produce L-theanine, and the results are shown in Fig. 7b. In the single-enzyme catalytic system, 63.9±1.5 g/L L-theanine was produced within 6 h after the addition of 450 mM ATP, while in the catalytic system of GMAS and *Ch*PPK_D82N-K103E_, the addition of 5 mM ATP was used to produce L-theanine. Under the ATP regeneration system of *Ch*PPK_D82N-K103E_, the yield of L-theanine reached 62.7±1.1 g/L with a conversion rate of 90.1%. In summary, the mutant enzyme was shown to replace the addition of the substrate ATP efficiently and similarly, greatly saving the reaction cost. The final yield of L-theanine could reach 62.7±1.1 g/L, with a high conversion rate of 90.1±1.6 %. As we know, the high toxicity of ethylamine requires that the residual amount of this substrate should be reduced as much as possible in the reaction. We tested the residual amount of ethylamine in the final reaction solution and found that the yields of ethylamine were still low as expected (0.29 g/L to 3.5 g/L) (Additional file 1: Table S4). This proved that giving the reaction an efficient ATP supply driven by the mutant enzyme enhanced the conversion rate of substrate to product, reduced the accumulation of toxic substrate and maximized the substrate value.

GGT was considered as the other pathway to catalyze the production of L-theanine (Fig. 7a). Although this enzyme does not require the expensive ATP (8007 $/kg), it needs the glutamine (560 $/kg) as a substrate. Yang et al. [4] could convert 175 g/L glutamine to 70 g/L L-theanine with a 67 % conversion rate by GGT enzymatic catalysis. This indicated that adding an excess of glutamine could also produce a high yield of L-theanine. Actually, glutamine was not cheap and we had sought an enzyme catalytic method for glutamine to make the enzymatic production more economical. Further, the pH of GS is about 8.0 and GGT is generally 10.0, the catalysis of GS and GGT seems not desirable. We performed glutamine production from glutamate with GS enzyme, and this process also requires ATP, so we preliminarily explored the production of glutamine under ATP efficient regeneration. Adding 150 mM ATP, 95.1±2.3 mM glutamine was generated in 4 h. Then the GS (6 U/mL) and *Ch*PPK_D82N-K103E_ (5 U/mL) reaction was carried out to obtain 94.4±1.44 mM glutamine in 4 h (Additional file 1: Table S4). They displayed the similar production of L-glutamine. After enhanced ATP cycle regeneration by mutant, the conversion rate of substrate (glutamate) was 94.4±1.4%, increased by 44.8 % compared with the wild *Ch*PPK enzyme. Therefore, on the pathway of L-theanine synthesis from glutamine, considering the cost and solving the problem of the expensive substrate ATP or glutamine, we prefer to synthesize L-theanine starting from glutamate (38.1 $/kg). In the one-step synthesis of L-theanine catalyzed by GMAS enzyme, we enhanced the recycling and regeneration of ATP by mutation, improved the conversion capacity of glutamate substrate, and finally obtained 62.7±1.1 g/L L-theanine in 200 mL system. At the same time, we also tried to synthesize glutamine from glutamate to reduce the cost of L-theanine production catalyzed by GGT enzyme. Finally, we obtained 13.8±0.2 g/L glutamine with the conservation rate of 94.4±1.4 %. Due to the difference in the optimal pH between GS and GGT, we will further target the enzymatic modification of GGT to drop the pH of this enzyme and better cascade the production of L-theanine.

## Conclusions

In this research, we displayed a high-throughput dual system screening with intracellular and extracellular. With the improvement of substrate spectrum and tolerance, the catalytic activity of *Ch*PPK_D82N-K103E_ was increased to 430.2% and was considered as the highest catalytic efficiency among the major polyphosphate kinases. In the cascade catalysis or in vitro biosynthesis system, in addition to preferentially selecting ATP with low energy consumption or avoiding the use of ATP, an ATP regeneration system with low cost, high activity and high yield is also one of the strategies of synthetic biology. This efficient ATP regeneration system can not only produce glutamine from glutamate to further produce L-theanine, but also directly produce L-theanine from glutamate in one step. Adding GMAS and *Ch*PPK_D82N-K103E_, L-theanine was produced by 62.7 ± 1.1 g/L with high conversation rate and low cost. To the best of our knowledge, this is a positively combination of high yield, high conversion rate and high economic value of enzyme catalysis. After achieving the best efficient PPK-mutant enzyme, the addition of *Ch*PPK_D82N-K103E_ can significantly improve the efficiency and sustainability of ATP regeneration, which will enhance the catalytic efficiency and save the cost in energy-demanding catalytic reactions.

In addition, we have constructed a co-expression system for GMAS-*Ch*PPK_D82N-K103E_ in *E. coli* and are currently optimizing this system for whole-cell catalytic L-theanine production as the simple form of catalysis. The sustainability of efficient ATP-regeneration demonstrated by *Ch*PPK_D82N-K103E_ will be further used in biocatalytic reactions to produce high-value food chemicals such as glutathione, SAM and so on. It will further be a biological reaction platform to strengthen the sustainable supply of ATP-regeneration in enzyme expression intensity, enzyme immobilization and other strategies.

### Supplementary Information

**Additional file 1:** **Fig. S1. **An evolutionary tree of PPK enzymes. **Fig. S2. a** Crude enzyme expression of PPK. Lane 1: marker; lane 2: BL_21_/28a; lane 3: BL_21_/28a-PaPPKI; lane 4: BL_21_/28a-PaPPKII; lane 5: BL_21_/28a-PaPPKIII; lane 6: BL_21_/28a-*Bl*PPK; lane 7: BL_21_/28a-*Cg*PPK; lane 8: BL_21_/28a-*Eb*PPK; lane 9: BL_21_/28a-*Ch*PPK; lane 10: marker; lane 11: BL_21_/pXMJ19; lane 12: BL_21_/pXMJ19-*Eb*PPK; lane 13: BL_21_/pXMJ19-*Ch*PPK. (The expression of *EB*PPK and *Ch*PPK on the 28a plasmid may be due to the fast translation of the proteins, the proteins were clustered together, and the expression of the two proteins were not clear by SDS-PAGE, see lane 8 and 9 in the **a**. Therefore, *EB*PPK and *Ch*PPK were chose to express on the pXMJ-19 plasmid.). **b** Pure enzyme expression of PPK. Lane 1: marker; lane 2-4: *Pa*PPKI; lane 5-6: *Pa*PPKII; lane 7: *Pa*PPKIII; lane 8: *Bl*PPK; lane 9: *Eb*PPK; lane 10: *Ch*PPK; lane 11: *Cg*PPK. **Fig. S3** **a** Absorbance values at different concentrations of TMB. **b** Absorbance values at different concentrations of H_2_O_2_. **c** Color diagram of TMB-ATP-H_2_O_2_. **Table S1. **Biocatalytic reactions involving ATP to produce high-value chemicals^a^. **Table S2.** The number of bases of the PPK protein. **Table S3.** The capacity and yield of PPK enzymes to produce ATP. **Table S4.** The ATP regeneration system to produce L-theanine and glutamine. **Table S5.** Strains and plasmids used in this work. **Table S6.** Primers used in this work.

## References

[1] Vu Huu K, Zangl R, Hoffmann J, Just A, Morgner N (2022). Bacterial F-type ATP synthases follow a well-choreographed assembly pathway. Nat Commun.

[2] Andexer JN, Richter M (2015). Emerging enzymes for ATP regeneration in biocatalytic processes. ChemBioChem.

[3] Lv QL, Hu MK, Tian LZ, Liu F, Wang Q, Xu MJ, Rao ZM (2021). Enhancing L-glutamine production in *Corynebacterium glutamicum* by rational metabolic engineering combined with a two-stage pH control strategy. Bioresour Technol.

[4] Yang TW, Liu SY, Liu HL, Long MF, Chen PC, Zhang X, Xu MJ, Rao ZM (2021). Semi-quantitative activity assays for high-throughput screening of higher activity gamma glutamyl transferase and enzyme immobilization to efficiently synthesize L-theanine. J Biotechnol.

[5] Zhang J, Quan C, Wang C, Wu H, Li Z, Ye Q (2016). Systematic manipulation of glutathione metabolism in *Escherichia coli* for improved glutathione production. Microb Cell Fact.

[6] Xiao Q, Niu J, Liu H, Liu Y, Zhou X (2019). High conversion of D-fructose into D-allulose by enzymes coupling with an ATP regeneration system. Mol Biotechnol.

[7] Fedorchuk TP, Khusnutdinova AN, Evdokimova E, Flick R, Di Leo R, Stogios P, Savchenko A, Yakunin AF (2019). One-pot biocatalytic transformation of adipic acid to 6-aminocaproic acid and 1,6-hexamethylenediamine using carboxylic acid reductases and transaminases. J Am Chem Soc.

[8] Chen H, Zhang YPJ (2021). Enzymatic regeneration and conservation of ATP: challenges and opportunities. Crit Rev Biotechnol.

[9] Alissandratos A, Caron K, Loan TD, Hennessy JE, Easton CJ (2016). ATP recycling with cell lysate for enzyme-catalyzed chemical synthesis, protein expression and PCR. ACS Chem Biol.

[10] Singh M, Tiwari P, Arora G, Agarwal S, Kidwai S, Singh R: Establishing virulence associated polyphosphate kinase 2 as a drug target for *Mycobacterium tuberculosis* (vol 6, 26900, 2016). Sci Rep-Uk 2020, 10.1038/srep26900 10.1038/srep26900 PMC4899718 27279366

[11] Brown MR, Kornberg A (2008). The long and short of it—polyphosphate, PPK and bacterial survival. Trends Biochem Sci.

[12] Campanella JR (1950). Structure and properties of the condensed phosphates. IV. Complex ion formation in polyphosphate solutions. J Am Chem Soc.

[13] Nocek B, Kochinyan S, Proudfoot M, Brown G, Evcokimova E, Osipiuk J, Edwards AM, Savchenko A, Joachimiak A, Yakunin AF (2008). Polyphosphate-dependent synthesis of ATP and ADP by the family-2 polyphosphate kinases in bacteria. P Natl Acad Sci USA.

[14] Lindner SN, Vidaurre D, Willbold S, Schoberth SM, Wendisch VF (2007). NCgl2620 encodes a class II polyphosphate kinase in *Corynebacterium glutamicum*. Appl Environ Microbiol.

[15] Mordhorst S, Singh J, Mohr MKF, Hinkelmann R, Keppler M, Jessen HJ, Andexer JN (2019). Several Polyphosphate Kinase 2 Enzymes Catalyse the Production of Adenosine 5'-Polyphosphates. ChemBioChem.

[16] Zhang HY, Ishige K, Kornberg A (2002). A polyphosphate kinase (PPK2) widely conserved in bacteria. P Natl Acad Sci USA.

[17] Motomura K, Hirota R, Okada M, Ikeda T, Ishida T, Kuroda A (2014). A new subfamily of polyphosphate kinase 2 (Class III PPK2) catalyzes both nucleoside monophosphate phosphorylation and nucleoside diphosphate phosphorylation. Appl Environ Microb.

[18] Tavanti M, Hosford J, Lloyd RC, Brown MJB (2021). ATP regeneration by a single polyphosphate kinase powers multigram-scale aldehyde synthesis in vitro. Green Chemistry.

[19] Zhu Y, Liang M, Li H, Ni H, Li L, Li Q, Jiang Z (2020). A mutant of Pseudoalteromonas carrageenovora arylsulfatase with enhanced enzyme activity and its potential application in improvement of the agar quality. Food Chem.

[20] Crowe J, Masone BS, Ribbe J (1996). One-step purification of recombinant proteins with the 6xHis tag and Ni-NTA resin. Mol Biotechnol.

[21] Bradford M (1976). A rapid and sensitive method for the quantitation of microgram quantities of protein utilizing the principle of protein-dye binding. Anal Biochem.

[22] Stanton RC (2012). Glucose-6-phosphate dehydrogenase, NADPH, and cell survival. IUBMB Life.

[23] Cui C, Ming H, Li L, Li M, Gao J, Han T, Wang Y (2020). Fabrication of an in-situ co-immobilized enzyme in mesoporous silica for synthesizing GSH with ATP regeneration. Mol Catal.

[24] Gottschalk J, Assmann M, Kuballa J, Elling L (2022). Repetitive synthesis of high-molecular-weight hyaluronic acid with immobilized enzyme cascades. Chemsuschem.

[25] Kaur J, Sharma R (2006). Directed evolution: an approach to engineer enzymes. Crit Rev Biotechnol.

[26] Shi Y, Tang M, Sun C, Pan Y, Liu L, Long Y, Zheng H (2019). ATP mimics pH-dependent dual peroxidase-catalase activities driving H_2_O_2_ decomposition. CCS Chemistry.

[27] Wang J, Wang X, Wang M, Bian Q, Zhong J (2022). Novel Ce-based coordination polymer nanoparticles with excellent oxidase mimic activity applied for colorimetric assay to organophosphorus pesticides. Food Chem.

[28] Zhang X, Yang Q, Lang Y, Jiang X, Wu P (2020). Rationale of 3,3',5,5'-Tetramethylbenzidine as the Chromogenic Substrate in Colorimetric Analysis. Anal Chem.

[29] Winkelman JT, Chandrangsu P, Ross W, Gourse RL (2016). Open complex scrunching before nucleotide addition accounts for the unusual transcription start site of *E. coli* ribosomal RNA promoters. Proc Natl Acad Sci U S A.

[30] Chiu J, March PE, Lee R, Tillett D (2004). Site-directed, Ligase-Independent Mutagenesis (SLIM): a single-tube methodology approaching 100% efficiency in 4 h. Nucleic Acids Res.

[31] Tong L, Zheng J, Wang X, Wang X, Huang H, Yang H, Tu T, Wang Y, Bai Y, Yao B (2021). Improvement of thermostability and catalytic efficiency of glucoamylase from *Talaromyces leycettanus* JCM12802 via site-directed mutagenesis to enhance industrial saccharification applications. Biotechnol Biofuels.

[32] Park H, Bradley P, Greisen P, Liu Y, Mulligan VK, Kim DE, Baker D, DiMaio F (2016). Simultaneous optimization of biomolecular energy functions on features from small molecules and macromolecules. J Chem Theory Comput.

[33] Lelievre CM, Balandras M, Petit JL, Vergne-Vaxelaire C, Zaparucha A (2020). ATP regeneration system in chemoenzymatic amide bond formation with thermophilic CoA ligase. ChemCatChem.

[34] Restiawaty E, Iwasa Y, Maya S, Honda K, Omasa T, Hirota R, Kuroda A, Ohtake H (2011). Feasibility of thermophilic adenosine triphosphate-regeneration system using *Thermus thermophilus* polyphosphate kinase. Process Biochem.

[35] Chen JJ, Zhu RS, Zhou JP, Yang TW, Zhang X, Xu MJ, Rao ZM (2021). Efficient single whole-cell biotransformation for L-2-aminobutyric acid production through engineering of leucine dehydrogenase combined with expression regulation. Bioresour Technol.

[36] Nocek BP, Khusnutdinova AN, Ruszkowski M, Flick R, Burda M, Batyrova K, Brown G, Mucha A, Joachimiak A, Berlicki L (2018). Structural insights into substrate selectivity and activity of bacterial polyphosphate kinases. Acs Catal.

