# Correction: A high-throughput dual system to screen polyphosphate kinase mutants for efficient ATP regeneration in l-theanine biocatalysis

**DOI:** 10.1186/s13068-023-02390-4

**Published:** 2023-09-15

**Authors:** Hui Gao, Mengxuan Li, Qing Wang, Tingting Liu, Xian Zhang, Taowei Yang, Meijuan Xu, Zhiming Rao

**Affiliations:** 1 The Key Laboratory of Industrial Biotechnology, Ministry of Education, School of Biotechnology, Jiangnan University, 1800 Lihu Road, Wuxi, 214122 Jiangsu China; 2 Yantai Shinho Enterprise Foods Co., Ltd., Yantai, 265503 China

**Correction: Biotechnology for Biofuels and Bioproducts (2023) 16:122** 10.1186/s13068-023-02361-9

Following publication of the original article [1], it came to the attention of the authors that the sequence of PPK12 was previously reported in an earlier publication [2] and registered under the UniProt ID: A0A3D5XRJ5. The sequence of EbPPK from this manuscript was registered under the Genbank ID: HCY06753.1. The protein sequence is of the same strain origin (*Erysipelotrichaceae bacterium*). However, ChPPK is the focus of research in this article and not EbPPK. The authors apologize for not being aware of the previous publication and have now cited the previous publication reporting the sequence [2] in the article. They have also cited [2] in the Results and Discussion section in relation to the fact that that EbPPK is better than ChPPK at regenerating ATP from AMP, which was reported by [2] previously.

The authors also state that the correction does not affect the discussion or conclusions and that they sincerely apologize for the unintentional errors.

The original article has been corrected.
