# Supplementary material for: A high-throughput dual system to screen polyphosphate kinase mutants for efficient ATP regeneration in L-theanine biocatalysis
Source: Biotechnol Biofuels Bioprod. 2023 Aug 3;16:122. doi: 10.1186/s13068-023-02361-9 (PMC10401862; doi:10.1186/s13068-023-02361-9)
Supplement: Supplementary file 1 — Additional file 1: Fig. S1. An evolutionary tree of PPK enzymes. Fig. S2. a Crude enzyme expression of PPK. Lane 1: marker; lane 2: BL21/28a; lane 3: BL21/28a-PaPPKI; lane 4: BL21/28a-PaPPKII; lane 5: BL21/28a-PaPPKIII; lane 6: BL21/28a-BlPPK; lane 7: BL21/28a-CgPPK; lane 8: BL21/28a-EbPPK; lane 9: BL21/28a-ChPPK; lane 10: marker; lane 11: BL21/pXMJ19; lane 12: BL21/pXMJ19-EbPPK; lane 13: BL21/pXMJ19-ChPPK. (The expression of EBPPK and ChPPK on the 28a plasmid may be due to the fast translation of the proteins, the proteins were clustered together, and the expression of the two proteins were not clear by SDS-PAGE, see lane 8 and 9 in the a. Therefore, EBPPK and ChPPK were chose to express on the pXMJ-19 plasmid.). b Pure enzyme expression of PPK. Lane 1: marker; lane 2-4: PaPPKI; lane 5-6: PaPPKII; lane 7: PaPPKIII; lane 8: BlPPK; lane 9: EbPPK; lane 10: ChPPK; lane 11: CgPPK. Fig. S3 a Absorbance values at different concentrations of TMB. b Absorbance values at different concentrations of H2O2. c Color diagram of TMB-ATP-H2O2. Table S1. Biocatalytic reactions involving ATP to produce high-value chemicalsa. Table S2. The number of bases of the PPK protein. Table S3. The capacity and yield of PPK enzymes to produce ATP. Table S4. The ATP regeneration system to produce L-theanine and glutamine. Table S5. Strains and plasmids used in this work. Table S6. Primers used in this work. [file 13068_2023_2361_MOESM1_ESM.docx]

**Additional file 1**

**A High Throughput Dual System to Screen Polyphosphate Kinase Mutants for Efficient ATP Regeneration in L-Theanine Biocatalysis**

Hui Gao^a^, Mengxuan Li^a^, Qing Wang^a^, Tingting Liu^b^, Xian Zhang^a^, Taowei Yang^a^, Meijuan Xu^*a^, Zhiming Rao^a^

^a^ The Key Laboratory of Industrial Biotechnology, Ministry of Education, School of Biotechnology, Jiangnan University, Wuxi, 214122, China

^b^ Yantai Shinho Enterprise Foods Co., Ltd., Yantai, 265503, China

*Corresponding author: Jiangnan University, School of Biotechnology, 1800 Li hu Road, Wuxi, Jiangsu 214122, China.

Tel: +86-510-85916881

Corresponding author Email address: xumeijuan@jiangnan.edu.cn (Meijuan Xu)


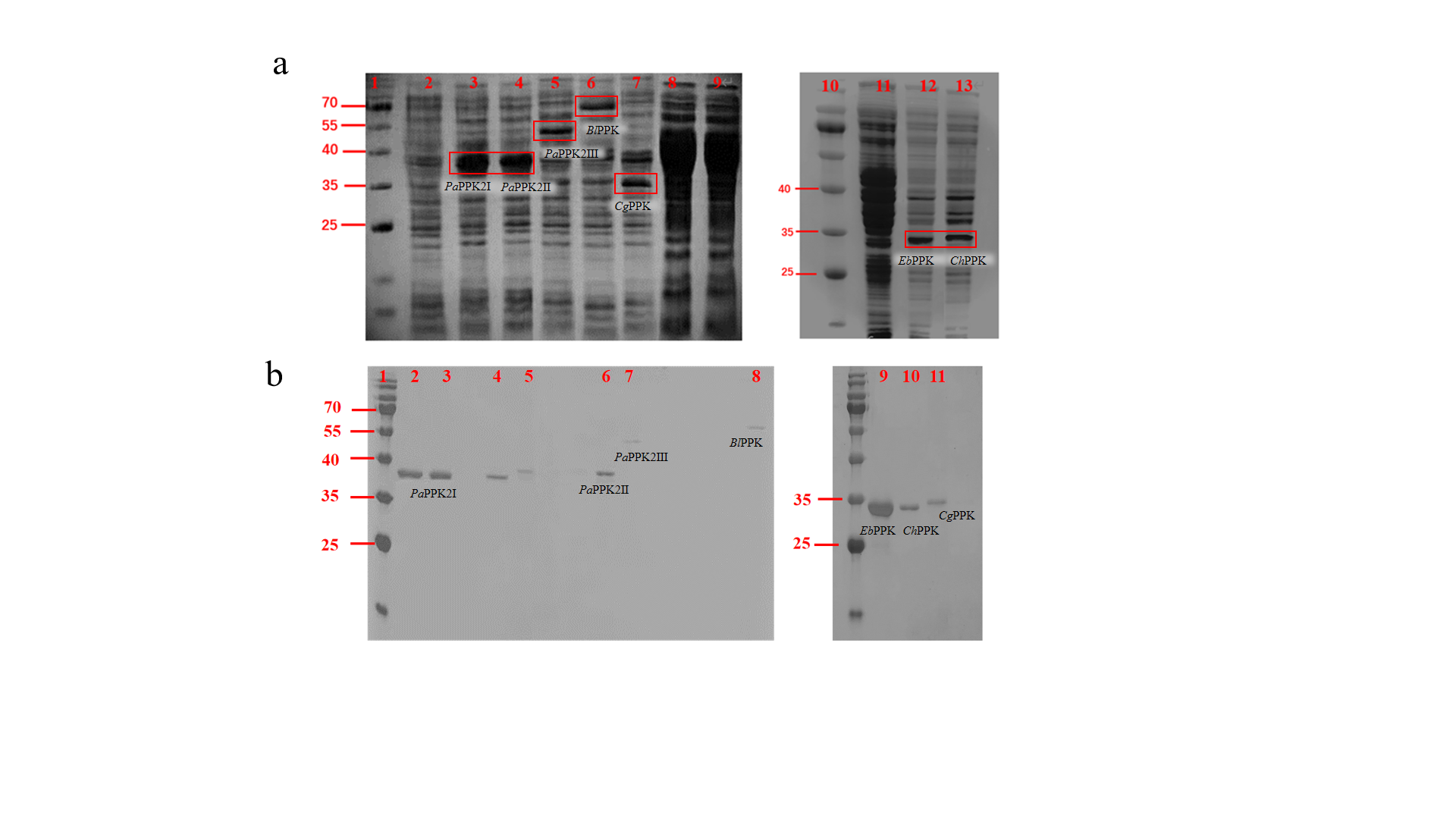

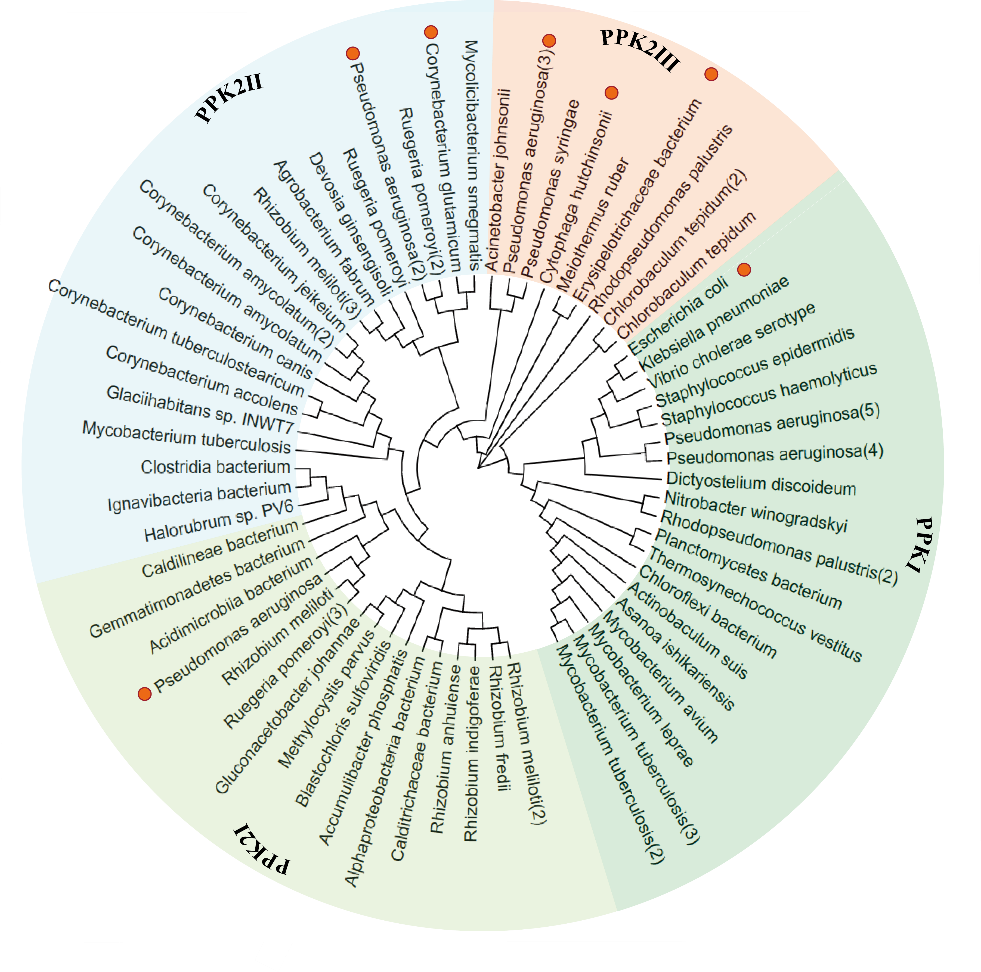
**Fig. S1** An evolutionary tree of PPK enzymes

**Fig. S2** (a) Crude enzyme expression of PPK. Lane 1: marker; lane 2: BL_21_/28a; lane 3: BL_21_/28a-PaPPKⅠ; lane 4: BL_21_/28a-PaPPKⅡ; lane 5: BL_21_/28a-PaPPKⅢ; lane 6: BL_21_/28a-*Bl*PPK; lane 7: BL_21_/28a-*Cg*PPK; lane 8: BL_21_/28a-*Eb*PPK; lane 9: BL_21_/28a-*Ch*PPK; lane 10: marker; lane 11: BL_21_/pXMJ19; lane 12: BL_21_/pXMJ19-*Eb*PPK; lane 13: BL_21_/pXMJ19-*Ch*PPK. (The expression of *EB*PPK and *Ch*PPK on the 28a plasmid may be due to the fast translation of the proteins, the proteins were clustered together, and the expression of the two proteins were not clear by SDS-PAGE, see lane 8 and 9 in the Fig. S2 a. Therefore, *EB*PPK and *Ch*PPK were chose to express on the pXMJ-19 plasmid.)

(b) Pure enzyme expression of PPK. Lane 1: marker; lane 2-4: *Pa*PPKⅠ; lane 5-6: *Pa*PPKⅡ; lane 7: *Pa*PPKⅢ; lane 8: *Bl*PPK; lane 9: *Eb*PPK; lane 10: *Ch*PPK; lane 11: *Cg*PPK.


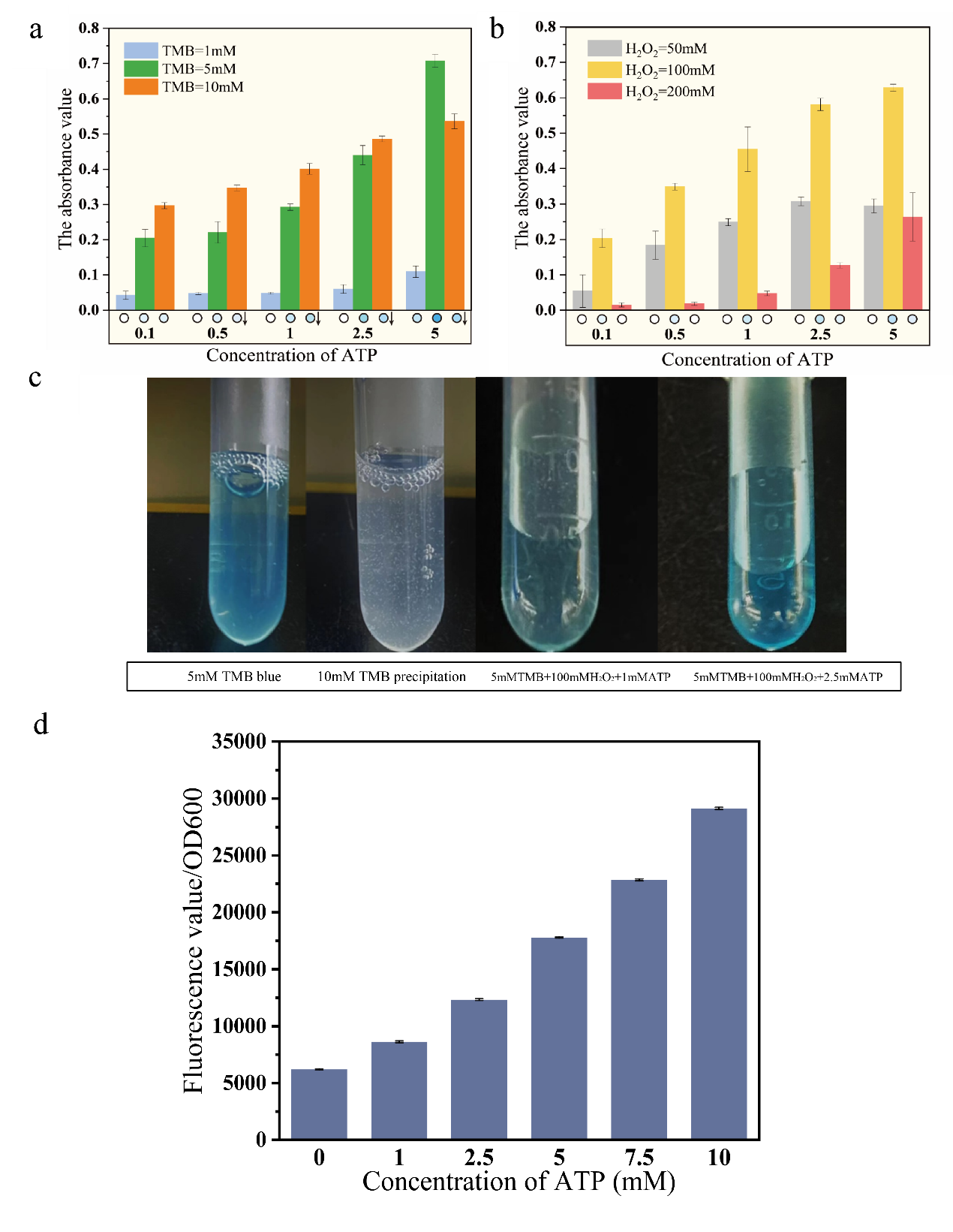
**Fig. S3** (a) Absorbance values at different concentrations of TMB. (b) Absorbance values at different concentrations of H_2_O_2._ (c) Color diagram of TMB-ATP-H_2_O_2_.


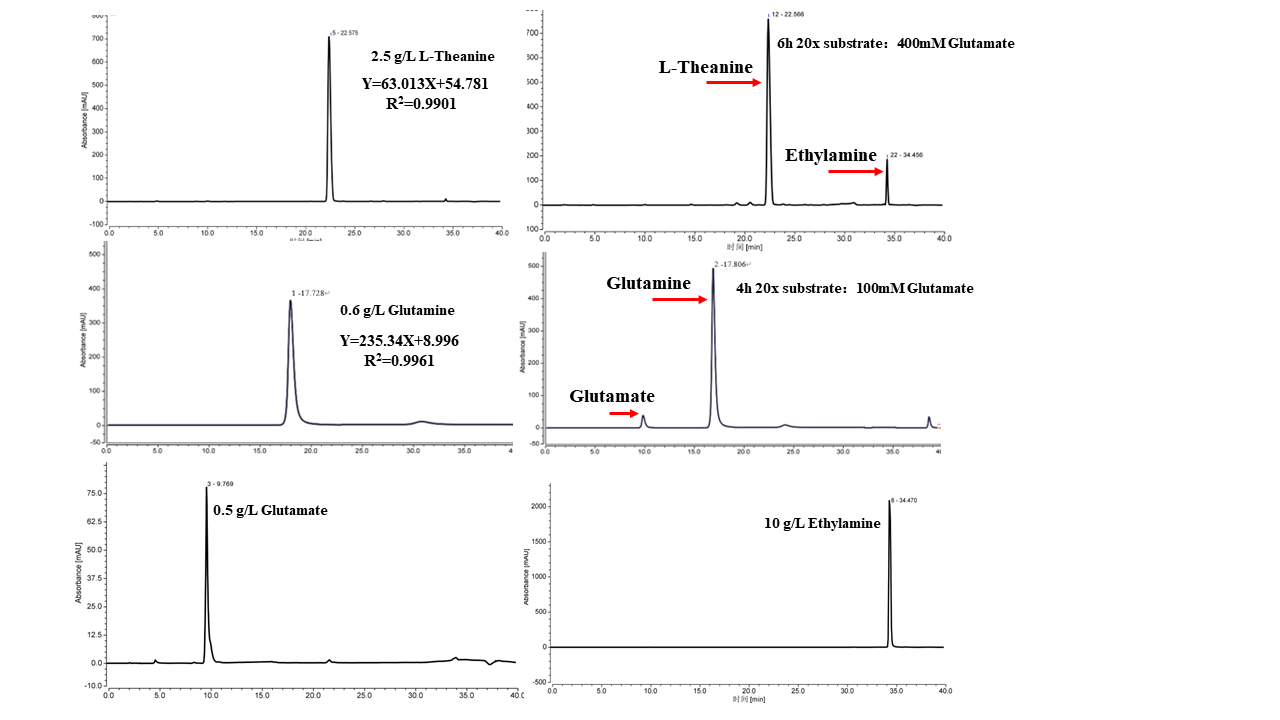
**The HPLC chromatogram：**

**The standard curve：**

The Standard curve for PPK enzyme activity assay: $y=3.3292x+0.0088 R^{2}=0.9978$ (x: concentration, mM; y: absorbance value)

**Table S1 Biocatalytic reactions involving ATP to produce high-value chemicals ^a^.**

| **Production** | | | **Enzyme** | | **Reaction** | |
| --- | --- | --- | --- | --- | --- | --- |
| Amino acid derivatives | L-Theanine | | GMAS (EC 6.3.4.12) | | Glutamate + ethylamine + ATP → L-Theanine + ADP | |
|  | Glutamine | | GS (EC 6.3.1.2) | | Glutamate + (NH_4_)_2_SO_4_ + ATP → Glutamine + ADP | |
|  | Glutathione | | GshF (EC 6.3.2.3) | | Glutamate + Glycine + Cysteine + 2ATP → Glutathione + 2ADP | |
|  | S-adenosylmethionine | | MAT (EC 2.5.1.6) | | L-methionine + ATP → S-adenosylmethionine | |
| Saccharides | D‑Allulose | | RhaB (EC 2.7.1.5) | | D‑Fructose + ATP → D-Allulose + ADP | |
| Carboxylic acid derivatives | Amides | | McbA (EC 3.5.1.137) | | methylamine/butylamine/xylamine/propargylamine + 1-acetyl-9Hβ-carboline-3-carboxylic acid + ATP → amides productions + AMP | |
|  | Aldehyde | | CARs (EC 1.2.99.6) | | Carboxylic acids +ATP+NADPH → Aldehyde | |
| Cofactors | Acyl-CoA | | Cat (EC 6.3.2.49) | | acetate/benzoate + CoA + ATP → Acyl-CoA + AMP | |
| Dipeptide  products | L-alanyl-L-glutamine | | YwfE (EC 6.3.2.49) | | L-alanine + L-glutamine + ATP → L-alanyl-L-glutamine + ADP | |
|  | D-alanyl-D-alanine | | Lal (EC 6.3.2.4) | | 2 D-alanine + ATP → ADP + D-alanyl-D-alanine | |
|  | Isoleucine-leucine | | NRPS ( EC 6.2.1.66) | | Isoleucine + Leucine + ATP → Isoleucine-leucine + ADP | |
|  | D-Alanine−D-alanine | | Ddl (E.C.6.3.2.4) | | 2 D-alanine + ATP → D-Alanine−D-alanine | |
|  |  | … | | … | | … |

^a^ The data were obtained from the BRENDA database.

**Table S2** **The number of bases of the PPK protein**

| Sources of the strain | Protein molecular weight |
| --- | --- |
| *Escherichia coli* (*Bl*PPK) | 70KDa |
| *Corynebacterium glutamicum* (*Cg*PPK) | 33KDa |
| *Pseudomonas aeruginosa* (*Pa*PPK2Ⅰ) | 38KDa |
| *Pseudomonas aeruginosa* (*Pa*PPK2Ⅱ) | 39KDa |
| *Pseudomonas aeruginosa* (*Pa*PPK2Ⅲ) | 55KDa |
| *Erysipelotrichaceae bacterium* (*Eb*PPK) | 33KDa |
| *Cytophaga hutchinsonii* (*Ch*PPK) | 34KDa |

**Table S3 The capacity and yield of PPK enzymes to produce ATP.**

| **Enzymes** | **ATP concentration**  **（g/L）** | **The space time yield of ATP**  **（g/L/h）** |
| --- | --- | --- |
|  |  |  |
| *Pa*PPK2Ⅰ | 6.8±0.12 | 1.70±0.03 |
| *Pa*PPK2Ⅱ | 4.71±0.24 | 1.18±0.06 |
| *Pa*PPK2Ⅲ | 5.5±0.19 | 1.38±0.05 |
| *Eb*PPK | 7.5±0.21 | 1.88±0.05 |
| *Bl*PPK | 3.2±0.23 | 0.53±0.04 |
| *Cg*PPK | 6.87±0.31 | 1.15±0.05 |
| *Ch*PPK | 8.3±0.22 | 2.08±0.06 |
| *Ch*PPK_D82N-K103E_ | 18.8±0.19 | 4.7±0.05 |

**Table S4 The ATP regeneration system to produce L-theanine and glutamine.**

| **Substrates** | **Enzyme** | **Production of L-theanine or glutamine**  **(g/L)** | **The amount of**  **ethylamine hydrochloride as residues (g/L)** | **Conversion rate (%)** |
| --- | --- | --- | --- | --- |
| 100 mM Glutamate  100 mM Ethylamine  150 mM ATP | GMAS | 16.8±1.3 | 0.29±0.15 | 96.4±7.5 |
| 100 mM Glutamate  100 mM Ethylamine  5 mM ATP | GMAS  *Ch*PPK | 10.8±0.9 | 2.9±0.42 | 62.1±5.2 |
| 100 mM Glutamate  100 mM Ethylamine  5 mM ATP | GMAS  *Ch*PPK_D82N-K103E_ | 16.6±0.79 | 0.34±0.22 | 95.6±4.5 |
| 200 mM Glutamate  200 mM Ethylamine  250 mM ATP | GMAS | 33.5±1.7 | 0.9±0.2 | 96.2±4.9 |
| 200 mM Glutamate  200 mM Ethylamine  5 mM ATP | GMAS  *Ch*PPK_D82N-K103E_ | 32.3±1.6 | 0.8±0.14 | 92.8±4.6 |
| 400 mM Glutamate  400 mM Ethylamine  450 mM ATP | GMAS | 63.9±1.5 | 3.5±0.4 | 91.8±2.2 |
| 400 mM Glutamate  400 mM Ethylamine  5 mM ATP | GMAS  *Ch*PPK_D82N-K103E_ | 62.7±1.1 | 2.9±1.1 | 90.1±1.6 |
| 100 mM Glutamate  100 mM (NH_4_)_2_SO_4_  150 mM ATP | GS | 13.9±0.33 | none | 95.1±2.3 |
| 100 mM Glutamate  100 mM (NH_4_)_2_SO_4_  5 mM ATP | GS  *Ch*PPK | 13.8±0.2 | none | 65.2±1.4 |
| 100 mM Glutamate  100 mM (NH_4_)_2_SO_4_  5 mM ATP | GS  *Ch*PPK_D82N-K103E_ | 13.8±0.2 | none | 94.4±1.4 |

**Table S5** **Strains and plasmids used in this work.**

| **Strains and plasmids** | **Relevant characteristic(s)** | **Source** |  |
| --- | --- | --- | --- |
| Strains |  |  |  |
| *E. coli* BL21(DE3) | *F-dcm ompT hsdS (rB-mB-) gal λ(DE3)* | Invitrogen |  |
| EB01 | *E. coli* BL21(DE3), expression *ppk* gene from *E. coli* BL21 in pET28a (+) plasmids | In this work | |
| EB02 | *E. coli* BL21(DE3), expression *ppk* gene from *C. glutamicum* ATCC13032 in pET28a (+) plasmids | In this work | |
| EB03 | *E. coli* BL21(DE3), expression *ppk2A* gene from *Pseudomonas aeruginosa* in pET28a (+) plasmids | In this work | |
| EB04 | *E. coli* BL21(DE3), expression *ppk2B* gene from *Pseudomonas aeruginosa* in pET28a (+) plasmids | In this work | |
| EB05 | *E. coli* BL21(DE3), expression *ppk2C* gene from *Pseudomonas aeruginosa* in pET28a (+) plasmids | In this work | |
| EB06 | *E. coli* BL21(DE3), expression *ppk* gene from *Erysipelotrichaceae bacterium* in pET28a (+) plasmids | In this work | |
| EB07 | *E. coli* BL21(DE3), expression *ppk* gene from *Cytophaga hutchinsonii* in pET28a (+) plasmids | In this work | |
| EB08 | *E. coli* BL21(DE3), expression *ppk* gene from *Erysipelotrichaceae bacterium* in pXMJ-19 plasmids | In this work | |
| EB09 | *E. coli* BL21(DE3), expression *ppk* gene from *Cytophaga hutchinsonii* in pXMJ-19 plasmids | In this work | |
| EB10 | *E. coli* BL21(DE3), expression *rrnB* P1-*gfp* gene in pXMJ-19 plasmids | In this work | |
| EB11 | *E. coli* BL21(DE3), expression *chppk* and *rrnB* P1-gfp gene in pXMJ-19 plasmids | In this work | |
| EB12 | *E. coli* BL21(DE3), expression *chppk*-muts and *rrnB* P1-gfp gene in pXMJ-19 plasmids | In this work | |
| EB13 | *E. coli* BL21(DE3), expression *glnA* gene from *Corynebacterium glutamicum* in pET28a (+) plasmids | In this work | |
| EB14 | *E. coli* BL21(DE3), expression *gmas* gene from *Methylovorus mays* in pET28a (+) plasmids | In this work | |
| D1 | *E. coli* BL21(DE3), expression *Ch*PPK_D82N_ in pXMJ-19 plasmids | In this work | |
| D2 | *E. coli* BL21(DE3), expression *Ch*PPK_D82H_ in pXMJ-19 plasmids | In this work | |
| D3 | *E. coli* BL21(DE3), expression *Ch*PPK_D82T_ in pXMJ-19 plasmids | In this work | |
| K1 | *E. coli* BL21(DE3), expression *Ch*PPK_K103E_ in pXMJ-19 plasmids | In this work | |
| K2 | *E. coli* BL21(DE3), expression *Ch*PPK_K103V_ in pXMJ-19 plasmids | In this work | |
| K3 | *E. coli* BL21(DE3), expression *Ch*PPK_K103G_ in pXMJ-19 plasmids | In this work | |
| K4 | *E. coli* BL21(DE3), expression *Ch*PPK_K103D_ in pXMJ-19 plasmids | In this work | |
| DK | *E. coli* BL21(DE3), expression *Ch*PPK_D82N-K103E_ in pXMJ-19 plasmids | In this work | |
| Plasmids |  |  | |
| pET-28a (+) | *E. coli* expression vector, Km^R^ | Laboratory | |
| pXMJ-19 | P*tac* promoter, Chl^R^ | Laboratory | |

**Table S6 Primers used in this work**

| F | TTAAGCTTGCATGCCTGCAAAAGGAGGACAACCATGGCAACCG |
| --- | --- |
| R | AGCTGAATTCGAGCTCGTTAGTGGTGGTGGTGGTGGTGATCGG |
| **103 sites** |  |
| E-F | TGACTTCCTTCGAAGTGCCATCCAAGATCGAACTGTCCCA |
| E-R | TGGCACTTCGAAGGAAGTCACCTTCACGCCTTGTG |
| A-F | TGACTTCCTTCGCAGTGCCATCCAAGATCGAACTGTCCCA |
| A-R | TGGCACTGCGAAGGAAGTCACCTTCACGCCTTGTG |
| D-F | TGACTTCCTTCGATGTGCCATCCAAGATCGAACTGTCCCA |
| D-R | TGGCACATCGAAGGAAGTCACCTTCACGCCTTGTG |
| F-F | TGACTTCCTTCTTCGTGCCATCCAAGATCGAACTGTCCCA |
| F-R | TGGCACGAAGAAGGAAGTCACCTTCACGCCTTGTG |
| G-F | TGACTTCCTTCGGCGTGCCATCCAAGATCGAACTGTCCCA |
| G-R | TGGCACGCCGAAGGAAGTCACCTTCACGCCTTGTG |
| I-F | TGACTTCCTTCATCGTGCCATCCAAGATCGAACTGTCCCA |
| I-R | TGGCACGATGAAGGAAGTCACCTTCACGCCTTGTG |
| L-F | TGACTTCCTTCCTGGTGCCATCCAAGATCGAACTGTCCCA |
| L-R | TGGCACCAGGAAGGAAGTCACCTTCACGCCTTGTG |
| N-F | TGACTTCCTTCAACGTGCCATCCAAGATCGAACTGTCCCA |
| N-R | TGGCACGTTGAAGGAAGTCACCTTCACGCCTTGTG |
| V-F | TGACTTCCTTCGTGGTGCCATCCAAGATCGAACTGTCCCA |
| V-R | TGGCACCACGAAGGAAGTCACCTTCACGCCTTGTG |
| W-F | TGACTTCCTTCTGGGTGCCATCCAAGATCGAACTGTCCCA |
| W-R | TGGCACCCAGAAGGAAGTCACCTTCACGCCTTGTG |
| Y-F | TGACTTCCTTCTACGTGCCATCCAAGATCGAACTGTCCCA |
| Y-R | TGGCACGTAGAAGGAAGTCACCTTCACGCCTTGTG |
| Q-F | TGACTTCCTTCCAAGTGCCATCCAAGATCGAACTGTCCCA |
| Q-R | TGGCACTTGGAAGGAAGTCACCTTCACGCCTTGTG |
| M-F | TGACTTCCTTCATGGTGCCATCCAAGATCGAACTGTCCCA |
| M-R | TGGCACCATGAAGGAAGTCACCTTCACGCCTTGTG |
| S-F | TGACTTCCTTCTCCGTGCCATCCAAGATCGAACTGTCCCA |
| S-R | TGGCACGGAGAAGGAAGTCACCTTCACGCCTTGTG |
| T-F | TGACTTCCTTCACCGTGCCATCCAAGATCGAACTGTCCCA |
| T-R | TGGCACGGTGAAGGAAGTCACCTTCACGCCTTGTG |
| C-F | TGACTTCCTTCGCAGTGCCATCCAAGATCGAACTGTCCCA |
| C-R | TGGCACTGCGAAGGAAGTCACCTTCACGCCTTGTG |
| P-F | TGACTTCCTTCCCAGTGCCATCCAAGATCGAACTGTCCCA |
| P-R | TGGCACTGGGAAGGAAGTCACCTTCACGCCTTGTG |
| H-F | TGACTTCCTTCCACGTGCCATCCAAGATCGAACTGTCCCA |
| H-R | TGGCACGTGGAAGGAAGTCACCTTCACGCCTTGTG |
| R-F | TGACTTCCTTCCGCGTGCCATCCAAGATCGAACTGTCCCA |
| R-R | TGGCACGCGGAAGGAAGTCACCTTCACGCCTTGTG |
| **82 sites** |  |
| E-F | CAGCCGGCAAGGAAGGCACCGTGAAGCACATCATGAC |
| E-R | TCACGGTGCCTTCCTTGCCGGCTGCATCCATTGCTTGGA |
| A-F | CAGCCGGCAAGGCAGGCACCGTGAAGCACATCATGAC |
| A-R | TCACGGTGCCTGCCTTGCCGGCTGCATCCATTGCTTGGA |
| G-F | CAGCCGGCAAGGGCGGCACCGTGAAGCACATCATGAC |
| G-R | TCACGGTGCCGCCCTTGCCGGCTGCATCCATTGCTTGGA |
| F-F | CAGCCGGCAAGTTCGGCACCGTGAAGCACATCATGAC |
| F-R | TCACGGTGCCGAACTTGCCGGCTGCATCCATTGCTTGGA |
| L-F | CAGCCGGCAAGCTGGGCACCGTGAAGCACATCATGAC |
| L-R | TCACGGTGCCCAGCTTGCCGGCTGCATCCATTGCTTGGA |
| I-F | CAGCCGGCAAGATCGGCACCGTGAAGCACATCATGAC |
| I-R | TCACGGTGCCGATCTTGCCGGCTGCATCCATTGCTTGGA |
| V-F | CAGCCGGCAAGGTGGGCACCGTGAAGCACATCATGAC |
| V-R | TCACGGTGCCCACCTTGCCGGCTGCATCCATTGCTTGGA |
| N-F | CAGCCGGCAAGAACGGCACCGTGAAGCACATCATGAC |
| N-R | TCACGGTGCCGTTCTTGCCGGCTGCATCCATTGCTTGGA |
| Y-F | CAGCCGGCAAGTACGGCACCGTGAAGCACATCATGAC |
| Y-R | TCACGGTGCCGTACTTGCCGGCTGCATCCATTGCTTGGA |
| W-F | CAGCCGGCAAGTGGGGCACCGTGAAGCACATCATGAC |
| W-R | TCACGGTGCCCCACTTGCCGGCTGCATCCATTGCTTGGA |
| C-F | CAGCCGGCAAGGCAGGCACCGTGAAGCACATCATGAC |
| C-R | TCACGGTGCCTGCCTTGCCGGCTGCATCCATTGCTTGGA |
| H-F | CAGCCGGCAAGCACGGCACCGTGAAGCACATCATGAC |
| H-R | TCACGGTGCCGTGCTTGCCGGCTGCATCCATTGCTTGGA |
| K-F | CAGCCGGCAAGAAGGGCACCGTGAAGCACATCATGAC |
| K-R | TCACGGTGCCCTTCTTGCCGGCTGCATCCATTGCTTGGA |
| M-F | CAGCCGGCAAGATGGGCACCGTGAAGCACATCATGAC |
| M-R | TCACGGTGCCCATCTTGCCGGCTGCATCCATTGCTTGGA |
| P-F | CAGCCGGCAAGCCAGGCACCGTGAAGCACATCATGAC |
| P-R | TCACGGTGCCTGGCTTGCCGGCTGCATCCATTGCTTGGA |
| Q-F | CAGCCGGCAAGCAAGGCACCGTGAAGCACATCATGAC |
| Q-R | TCACGGTGCCTTGCTTGCCGGCTGCATCCATTGCTTGGA |
| R-F | CAGCCGGCAAGCGCGGCACCGTGAAGCACATCATGAC |
| R-R | TCACGGTGCCGCGCTTGCCGGCTGCATCCATTGCTTGGA |
| S-F | CAGCCGGCAAGTCCGGCACCGTGAAGCACATCATGAC |
| S-R | TCACGGTGCCGGACTTGCCGGCTGCATCCATTGCTTGGA |
| T-F | CAGCCGGCAAGACCGGCACCGTGAAGCACATCATGAC |
| T-R | TCACGGTGCCGGTCTTGCCGGCTGCATCCATTGCTTGGA |
